# *Spiclypeus shipporum* gen. et sp. nov., a Boldly Audacious New Chasmosaurine Ceratopsid (Dinosauria: Ornithischia) from the Judith River Formation (Upper Cretaceous: Campanian) of Montana, USA

**DOI:** 10.1371/journal.pone.0154218

**Published:** 2016-05-18

**Authors:** Jordan C. Mallon, Christopher J. Ott, Peter L. Larson, Edward M. Iuliano, David C. Evans

**Affiliations:** 1 Palaeobiology, Canadian Museum of Nature, PO Box 3443 Station “D”, Ottawa, Ontario, K1P 6P4, Canada; 2 Independent Researcher, PO Box 1515, Appleton, Wisconsin, 54912, United States of America; 3 Black Hills Institute, 217 Main Street, Hill City, South Dakota, 57745, United States of America; 4 Kadlec Medical Center, 888 Swift Boulevard, Richland, Washington, 99352, United States of America; 5 Department of Natural History, Royal Ontario Museum, 100 Queen’s Park, Toronto, Ontario, M5S 2C6, Canada; Monash Institute of Pharmaceutical Sciences, AUSTRALIA

## Abstract

This study reports on a new ceratopsid, *Spiclypeus shipporum* gen et sp. nov., from the lower Coal Ridge Member of the Judith River Formation in Montana, USA, which dates to ~76 Ma (upper Campanian). The species is distinguished by rugose dorsal contacts on the premaxillae for the nasals, laterally projecting postorbital horncores, fully fused and anteriorly curled P1 and P2 epiparietals, and a posterodorsally projecting P3 epiparietal. The holotype specimen is also notable for its pathological left squamosal and humerus, which show varied signs of osteomyelitis and osteoarthritis. Although the postorbital horncores of *Spiclypeus* closely resemble those of the contemporaneous ‘*Ceratops*’, the horncores of both genera are nevertheless indistinguishable from those of some other horned dinosaurs, including *Albertaceratops* and *Kosmoceratops*; ‘*Ceratops*’ is therefore maintained as a nomen dubium. Cladistic analysis recovers *Spiclypeus* as the sister taxon to the clade *Vagaceratops* + *Kosmoceratops*, and appears transitional in the morphology of its epiparietals. The discovery of *Spiclypeus* adds to the poorly known dinosaur fauna of the Judith River Formation, and suggests faunal turnover within the formation.

## Introduction

Ceratopsidae is a clade of megaherbivorous dinosaurs that arose during the Late Cretaceous and rapidly diversified in Asia and North America to become one of the most speciose dinosaur groups of their time [1]. Ceratopsids are most easily distinguished by their horned crania and expansive parietosquamosal frills, which were typically ornamented for display [2]. The two subfamilies, Centrosaurinae and Chasmosaurinae, primarily differ in the morphology of the horns and frill. Centrosaurines usually possess an enlarged nasal horncore or boss, abbreviated postorbital horncores, and a short and elaborately ornamented frill. Chasmosaurines typically have a short nasal horncore, elongate postorbital horncores, and a long frill with more conservative ornamentation.

The first recognized ceratopsid, ‘*Ceratops montanus*’ [3], was found in terrestrial deposits of the Campanian Judith River Formation (JRF) of Montana. Described on the basis of a pair of postorbital horncores and an occipital condyle, the species has long been considered a nomen dubium, owing to the non-diagnostic nature of these elements [1]. Many other named ceratopsids from the JRF (e.g., *Dysganus bicarinatus*, *D*. *encaustus*, *D*. *haydenanius*, *D*. *peiganus*, *Monoclonius crassus*, *M*. *recurvicornis*, *M*. *fissus*, *M*. *sphenocerus*) are also considered taxonomically invalid [1]. Presently, five valid ceratopsid species are recognized from the JRF: the centrosaurines *Avaceratops* [4] and *Albertaceratops* [5], and the chasmosaurines *Judiceratops* [6], *Medusaceratops* [7], and *Mercuriceratops* [8]. The time-equivalent Belly River Group in Alberta has been sampled over a similar period of time, yielding ~14 ceratopsid species [9]. The ceratopsid fauna of the JRF is therefore comparatively poorly understood.

Here we report on a new chasmosaurine from the JRF, distinguished, in part, by the dorsolaterally projecting postorbital horncores and conjointly fused, anteriorly curled epiossifications straddling the midline of the frill. The holotype exhibits several chronic pathologies, most notably on the squamosal and humerus, which attest to the resilience of the animal. The new species clarifies the evolutionary acquisition of the anteriorly curling epiossifications in the closely related *Kosmoceratops* and *Vagaceratops*, and further informs our understanding of both the biostratigraphy of the JRF and faunal provincialism on the Late Cretaceous continent of Laramidia.

## Geologic and Taphonomic Setting

**Fig 1:**
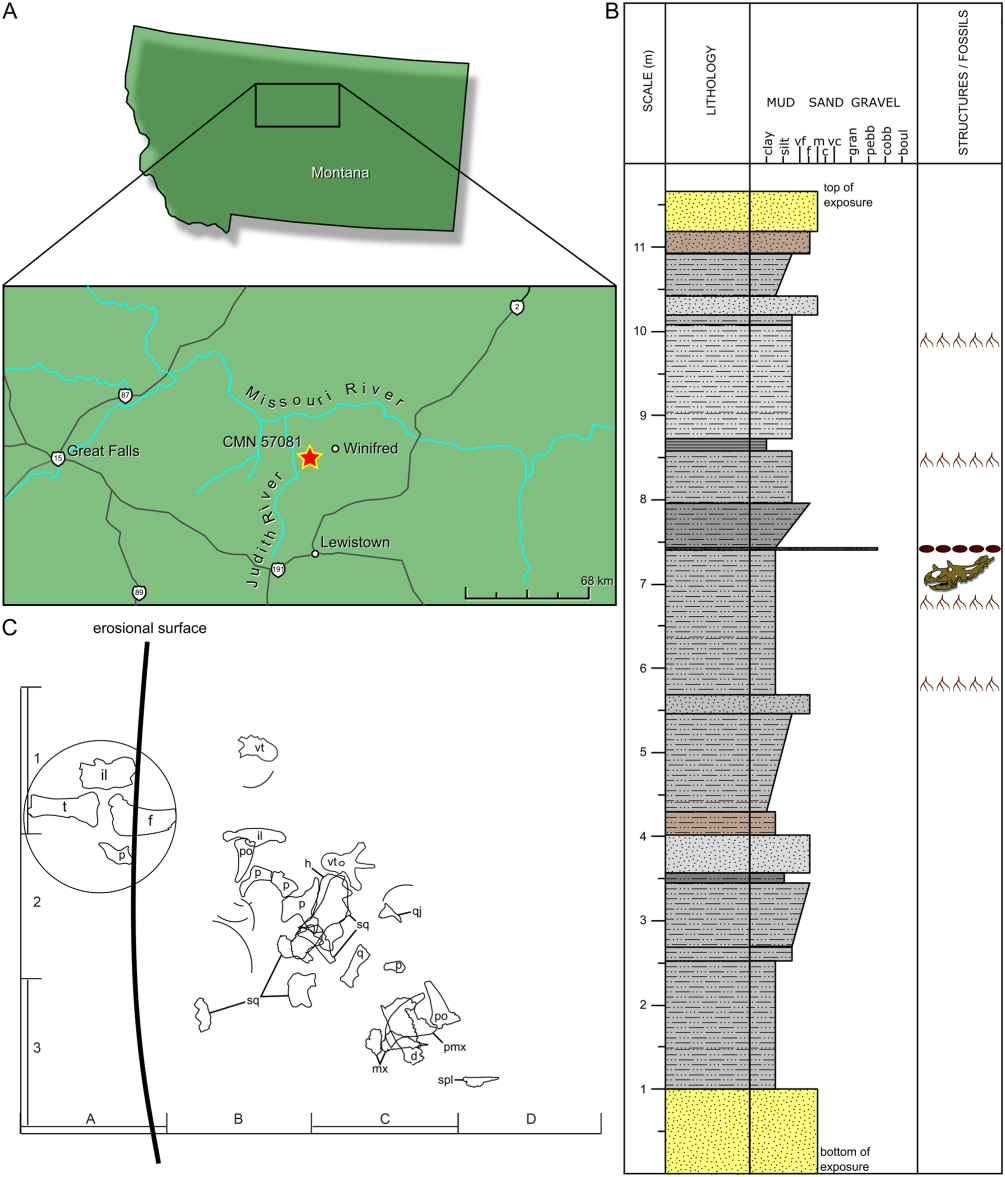
Locality data for the holotype of *Spiclypeus shipporum* gen et sp. nov. (CMN 57081). (A) Location of quarry within Montana, marked by star (map source: Google Earth); (B) stratigraphic section of holotype locality; (C) quarry map (by J. Small). Circled elements in (C) were exposed at the surface upon discovery. Curved lines in (C) represent ribs. Each grid square = 1 m^2^.

The holotype locality (Fig 1A) is in Fergus County, Montana, ~8 km WSW of the town of Winifred, in Section 1, Township 20N, Range 17E. GPS coordinates and photographs of the quarry are reposited with the specimen at the CMN.

**Fig 2:**
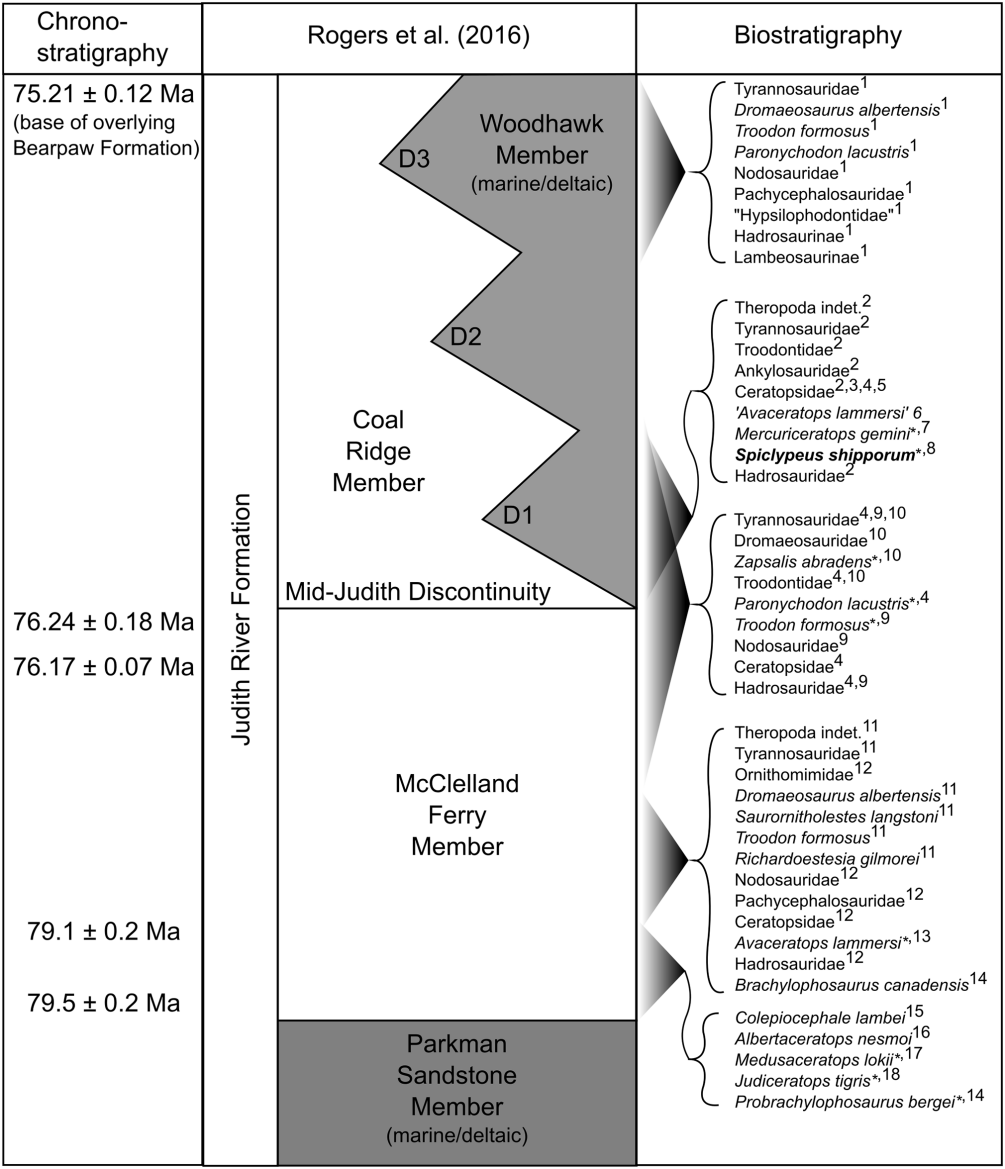
Idealized dinosaur biostratigraphy of the Judith River Formation. Dates and stratigraphic nomenclature from Rogers et al. [10]. Faunal data from: 1, Sahni [12]; 2, Rogers and Brady [13]; 3, Marsh [3]; 4, Cope [14]; 5, Cope [15]; 6, Penkalski and Dodson [16]; 7, Ryan et al. [8]; 8, this study; 9, Leidy [17]; 10, Cope [18]; 11, Fiorillo and Currie [19]; 12, Fiorillo [20]; 13, Dodson [4]; 14, Freedman et al. [21]; 15, Schott et al. [22]; 16, Ryan [5]; 17, Ryan et al. [7]; 18, Longrich [6]. All taxa from terrestrial McClelland Ferry or Coal Ridge members. Asterisk (*) denotes holotype of key species. Abbreviations: D1-3, disconformities bounding depositional sequences of Woodhawk Member.

The holotype specimen (CMN 58071) was collected from terrestrial deposits within the lower Coal Ridge Member of the Upper Cretaceous JRF [10] (Fig 2). The host stratum is mudstone, with abundant plant fragments throughout, and a siderite nodule zone at the top. This mudstone is interpreted as an overbank deposit in a floodplain environment. The measured section at the quarry (Fig 1B) spans ~11 m of outcrop. The lithologies within the measured section, including bentonitic mudstones, highly carbonaceous layers, and layers with high concentrations of siderite nodules, are consistent with the transgressive alluvial suite described by Rogers [11].

The contact between the JRF and the overlying Bearpaw Formation is exposed 2 km east of the quarry, ~80 m higher in section. Regionally, this contact dates to 75.21 ± 0.12 Ma, based on ^40^Ar/^39^Ar dating of a bentonite layer that occurs ~5 m above the contact near reference section 91-JRT-12 of Rogers et al. [10]. The Judith River-Claggett formational contact is ~95 m below the quarry, based on nearby well log data. The “mid-Judith discontinuity” [10], which is interpreted to coincide with the onset of transgression of the Bearpaw Sea, occurs 89 m above the Judith River-Claggett formational contact in reference section 91-JRT-8, ~27 km NNE of the quarry. This places the discontinuity at least 6 m below the quarry, in the subsurface. ^40^Ar/^39^Ar dating of a bentonite layer 10 m below the discontinuity, in reference section 91-JRT-8, yields an age of 76.24 Ma ± 0.18 [10]. CMN 58071 is therefore bracketed between ~76.24 Ma (mid-Judith discontinuity) and ~75.21 Ma (Judith-Bearpaw formational contact). The close proximity of the holotype to the mid-Judith discontinuity suggests that the specimen is much closer to 76 Ma in age (Fig 2). CMN 58071 is ~3 million years younger than the fauna at Kennedy Coulee, which includes the chasmosaurines *Medusaceratops lokii* and *Judiceratops tigris* ([6–7, 23]; see [10] for recalibrated dates). The holotype is approximately equivalent in age to the lower portion (Megaherbivore Assemblage Zone 1) of the Dinosaur Park Formation [24, 25], which includes the ceratopsids *Centrosaurus apertus* and *Mojoceratops perifania*. Comparison with radiometric dates from the Kaiparowits Formation of Utah indicates that CMN 57081 is also approximately equivalent in age to *Kosmoceratops richardsoni*, *Utahceratops gettyi*, and their associated fauna [26–28].

The holotype was badly broken and scattered prior to burial (Fig 1C). Larger bones, including the squamosals, parietal, and ilium, were broken into several fragments, which were discovered separated from each other by up to 2 m during excavation of the quarry. Many rib fragments exhibit oblique and/or transverse fractures, with sawtooth edges (sensu Shipman [29]). This is consistent with the interpretation that the elements still retained their collagen, and were broken prior to fossilization [30, 31]. Two tyrannosaurid teeth were found in the quarry, and parallel scratches that are consistent with tooth marks occur on several elements of the skeleton (e.g., partial ribs, fragment of nasal). It appears that the specimen was scavenged prior to burial, and some of the fractures may be due to trampling [32]. There is no preferred orientation or stream rounding to the bones (Fig 1C), which indicates that current transport was not a significant factor. There also appears to be no surface degradation to the bones, which indicates that the remains of this animal were rapidly buried after they were scavenged [32].

## Materials and Methods

### Collection, preparation, and description

CMN 57081 was discovered on private land in September of 2005 by Dr. B. D. Shipp and J. C. Gilpatrick. The specimen was eroding out of the side of an incised creek valley. Initially, fragments of the femur and ribs were surface collected. The remainder of the specimen was excavated manually by Dr. Shipp, J. Small, and team, and was removed over the following two years, using standard plaster and burlap jacketing techniques, with aluminum foil used as a separator. Preparation of the specimen was accomplished by White River Preparium (Hill City, South Dakota) with air scribes and air abrasive machines. Abrasives used include sodium bicarbonate for general preparation, and crushed marble for areas that were covered with ironstone concretion. Consolidants and adhesives used during preparation and restoration include polyvinyl butyral, and cyanoacrylate glues of varying viscosities. Infill of cracks and reconstruction of individual parts were done with two-part epoxy putty. All consolidants, glues, and epoxy putties are removable if necessary. Molds and casts were made of all cranial elements, and a complete skull was reconstructed (S1 File). Missing elements were restored using cast parts from a small *Triceratops horridus* skull at the Black Hills Institute (Hill City, South Dakota). Teeth were molded using President regular body polyvinylsiloxane (Coltène/Whaledent), and cast using Epotek 301 two-part epoxy. Dental microwear was imaged using an FEI XL30 environmental scanning electron microscope. The humerus of CMN 57081 was CT scanned at Kadlec Regional Medical Center (Richland, Washington) using a GE Medical Systems LightSpeed 16 scanner.

No permits were required for the described study, which complied with all relevant regulations. The holotype specimen is publicly accessible in the permanent repository of the CMN.

### Skeletochonology

In order to assess the ontogenetic age of CMN 57081, thin sections of the left fibula and a rib fragment were made using standard palaeohistological techniques [33]. Thin sections were made and imaged at the ROM Palaeohistology Laboratory. Prior to sectioning, the elements were moulded and casted, and casts accessioned into the collections at the ROM and the CMN. The elements were first embedded in Castolite resin, before being cut on a Buehler IsoMet 1000 Precision Cutter low-speed saw and mounted to a 2 mm plexiglass slide with either PSI 122/124 resin (fibula) or Scotch-Weld SF-100 cyanoacrylate (rib). The stubs were then cut down on the IsoMet saw, and subsequently ground down to the appropriate thickness using the grinding cup on a Hillquist Thin Section Machine, and finished by hand on a glass plate with 1000 silicon carbide grit. The slides were then imaged using a Nikon DS-Fi1 camera mounted to a Nikon AZ-100 microscope under plain-polarized and cross-polarized light. Images were processed and assembled using Nikon NIS-Elements (Basic Research) v. 3.13 imaging software. Both sections are on file at the CMN, but only the fibula section is described here.

### Phylogenetic analysis

To determine the evolutionary relationships of CMN 57081 within Chasmosaurinae, we performed cladistic analyses using maximum parsimony inference. We assessed the two character matrices of Brown and Henderson [34], which are modified from previous matrices by Sampson et al. [35] and Mallon et al. [36]. The two matrices assume different epiparietal homologies. The first homology scheme assumes the traditional hypothesis whereby a single median epiparietal is deemed ‘P0’ and all other epiparietals are given increasingly higher enumerations laterally. The second scheme assumes that the traditional P0 epiparietal is homologous to the medialmost, paired epiparietals on the dorsal surface of the frill (seen in *Anchiceratops*, *Pentaceratops*, and *Utahceratops*), which are positionally homologous to the single P0 epiparietal of *Regaliceratops*. Each matrix included 172 characters and 29 taxa. CMN 57081 was coded for 116 characters (67% of the total). We further modified the matrices as follows: (1) character 24 (presence/absence of forked distal end of posteroventral process of premaxilla) was recoded as ‘present’ for *Vagaceratops*; (2) character 49 (curvature of postorbital horncore in anterior view) was recoded as ‘laterally curved’ for *Kosmoceratops* (previously miscoded as ‘straight’); (3) character 69 (length of squamosal relative to parietal) was recoded as ‘squamosal slightly shorter than parietal, posterolateral-most margin of frill formed by the parietal’ for *Vagaceratops*; (4) character 70 (squamosal forms part of posterior margin of frill) was recoded as ‘absent’ for *Vagaceratops*; (5) characters 76–78 (relating to the shape of the posterior parietal bar) were recoded for *Chasmosaurus belli*, *C*. *russelli*, and *Vagaceratops* following Campbell et al. [37]; (6) character 100 (episquamosal S2 shape) was recoded as ‘low raised D‐shaped process’ for *Vagaceratops*; (7) character 103 (marginal ossification crossing squamosal‐parietal contact) was recoded as ‘absent’ for *Vagaceratops*, and as ‘absent’ for *Anchiceratops* (the previously coded polymorphism for *Anchiceratops* arguably reflects an ontogenetic transformation, which is inadvisable; see Hennig [38]); (8) character 104 (shape of marginal ossification crossing squamosal‐parietal contact) was recoded as ‘-‘ (not applicable) for *Vagaceratops*; (9) character 105 (number of epiparietals per side) was recoded as ‘five or more’ for *Vagaceratops*, and as polymorphic for *Chasmosaurus belli*; (10) codings for character 111 (presence/absence of epiparietal P1) were corrected in the traditional homology matrix (*Pachyrhinosaurus* was recoded as ‘absent’, chasmosaurines mistakenly coded as ‘absent’ were instead coded as ‘present’) (11) characters 112–114 (relating to the morphology of epiparietal 1) were recoded as ‘-‘ (not applicable) for *Albertaceratops* and *Pachyrhinosaurus* because epiparietal P1 is missing in these taxa; (12) postcranial characters 163–165 and 172 were coded for *Vagaceratops* (after description of Holmes [39]). Character 33 (adult nasal ornamentation type) was specified as ordered. Character 93 (presence/absence of marginal undulations of frill) was excluded because it is non-independent of character 95 (presence/absence of frill epiossifications). The original matrix files are available as supporting information (S2 and S3 Files).

The parsimony analysis was conducted using PAUP* 4.0b10 for Windows [40]. We used the branch and bound search algorithm, with *Leptoceratops* defined as the outgroup. Bootstrap values were estimated using 1,000 replicates and a random seed of 0. Bremer decay indices were calculated using TreeRot version 3 [41].

### Nomenclatural acts

The electronic edition of this article conforms to the requirements of the amended International Code of Zoological Nomenclature (ICZN), and hence the new names contained herein are available under that Code from the electronic edition of this article. This published work and the nomenclatural acts it contains have been registered in ZooBank, the online registration system for the ICZN. The ZooBank LSIDs (Life Science Identifiers) can be resolved and the associated information viewed through any standard web browser by appending the LSID to the prefix “http://zoobank.org/”. The LSID for this publication is: urn:lsid:zoobank.org:pub:0C3F47A8-FAC7-4340-9F41-52A9E12289C8. The electronic edition of this work was published in a journal with an ISSN, and has been archived and is available from the digital repositories PubMed Central and LOCKSS.

## Results

### Systematic palaeontology

Dinosauria Owen, 1842

Ornithischia Seeley, 1887

Ceratopsia Marsh, 1888

Neoceratopsia Sereno, 1986

Ceratopsidae Marsh, 1888

Chasmosaurinae Lambe, 1915

*Spiclypeus* gen. nov.

urn:lsid:zoobank.org:act:4F4B9688-15E9-43D0-9470-26C967D83316

#### Diagnosis

Monotypic, as for species.

*Spiclypeus shipporum*, gen. et sp. nov.

urn:lsid:zoobank.org:act:8A99EE07-DDD5-4726-BF05-88DA00EFF9BF

#### Etymology

The genus name (pronounced ‘spick-LIP-ee-us’) derives from the Latin for spike (*spica*) and shield (*clypeus*), referring to the many large, spike-like epiossifications about the margin of the parietosquamosal frill. The specific epithet (pronounced ‘ship-OR-um’) honours Dr. Bill and Linda Shipp, the original owners of the holotype, and their family.

#### Holotype

CMN 57081, a partial skull and postcranium.

#### Locality, horizon, and age

The holotype and only known specimen is from Fergus County, Montana, near the town of Winifred. The type locality occurs within the lower Coal Ridge Member of the JRF, several meters above the mid-Judith discontinuity, which dates to 76.24 Ma ± 0.18 Ma [10]. The top of the member has been dated to 75.21 ± 0.12 Ma [10], which marks the upper age bracket of the species.

#### Diagnosis

Chasmosaurine ceratopsid with autapomorphic rugose nasal contact on the lateral surface of the dorsal process of the premaxilla. *Spiclypeus* is also diagnosed by the following unique combination of characters: (1) postorbital horncores project dorsolaterally; (2) all six epiparietals fused at bases; (3) epiparietals P1 and P2 curl anteriorly from posterior margin of frill; (4) epiparietal P3 projects posterodorsally.

With respect to other chasmosaurines from the Judith River Formation (i.e., *Judiceratops*, *Medusaceratops*, *Mercuriceratops*), *Spiclypeus* can further be distinguished by the large, triangular epiossifications laterally on the parietal and squamosal. It also differs from *Judiceratops* [6] in the medial embayment of the posterior parietal bar. *Spiclypeus* lacks the laterally directed epiparietals that characterize *Medusaceratops* [7], and the hatchet-shaped lateral margin of the squamosal that characterizes *Mercuriceratops* [8].

### Description of CMN 57081

#### General comments about the skull

**Fig 3:**
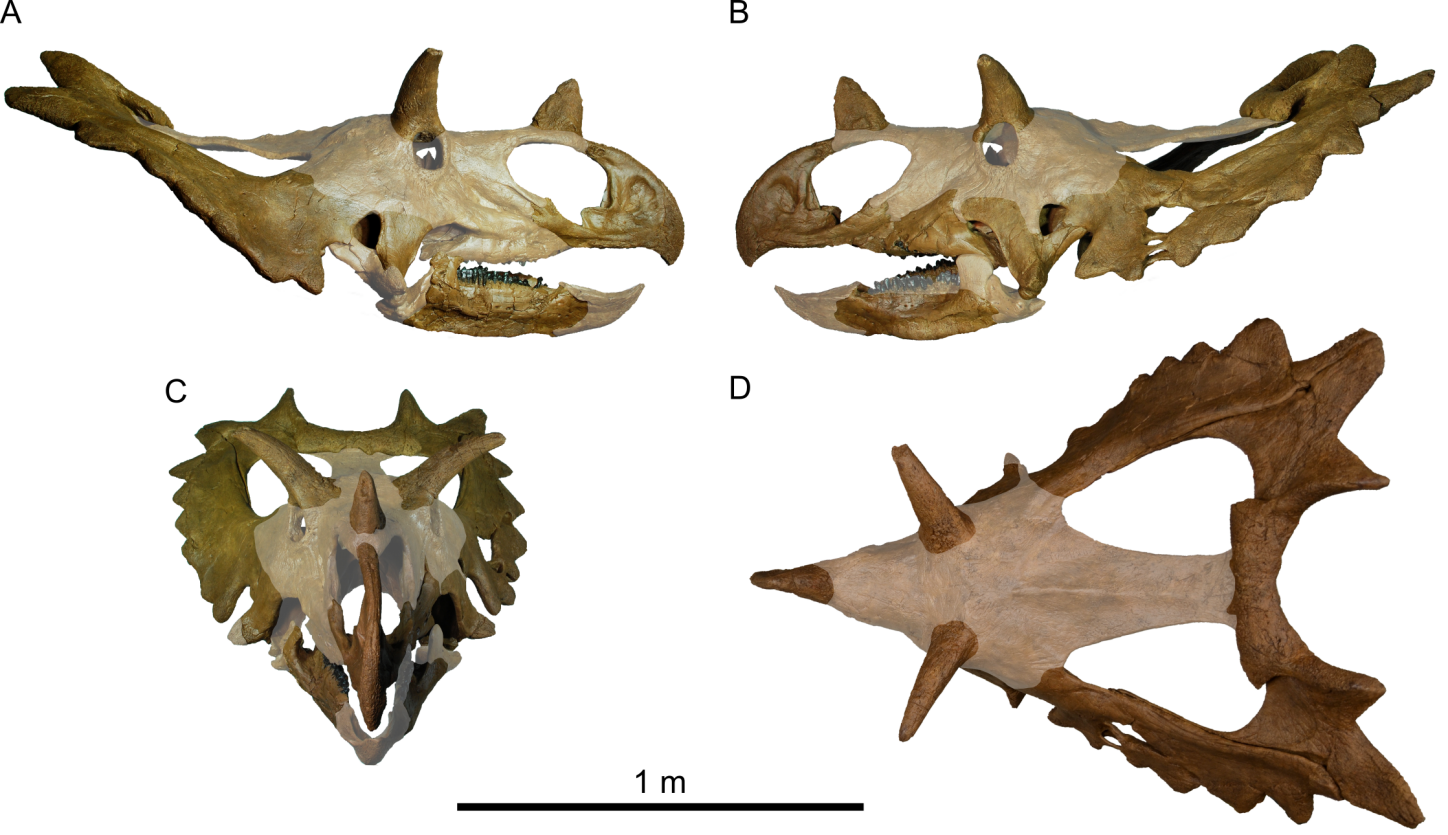
Skull reconstruction of *Spiclypeus shipporum* gen et sp. nov. (CMN 57081). (A) Left lateral view; (B) right lateral view; (C) anterior view; (D) dorsal view. Missing parts of skull shown faded.

**Fig 4:**
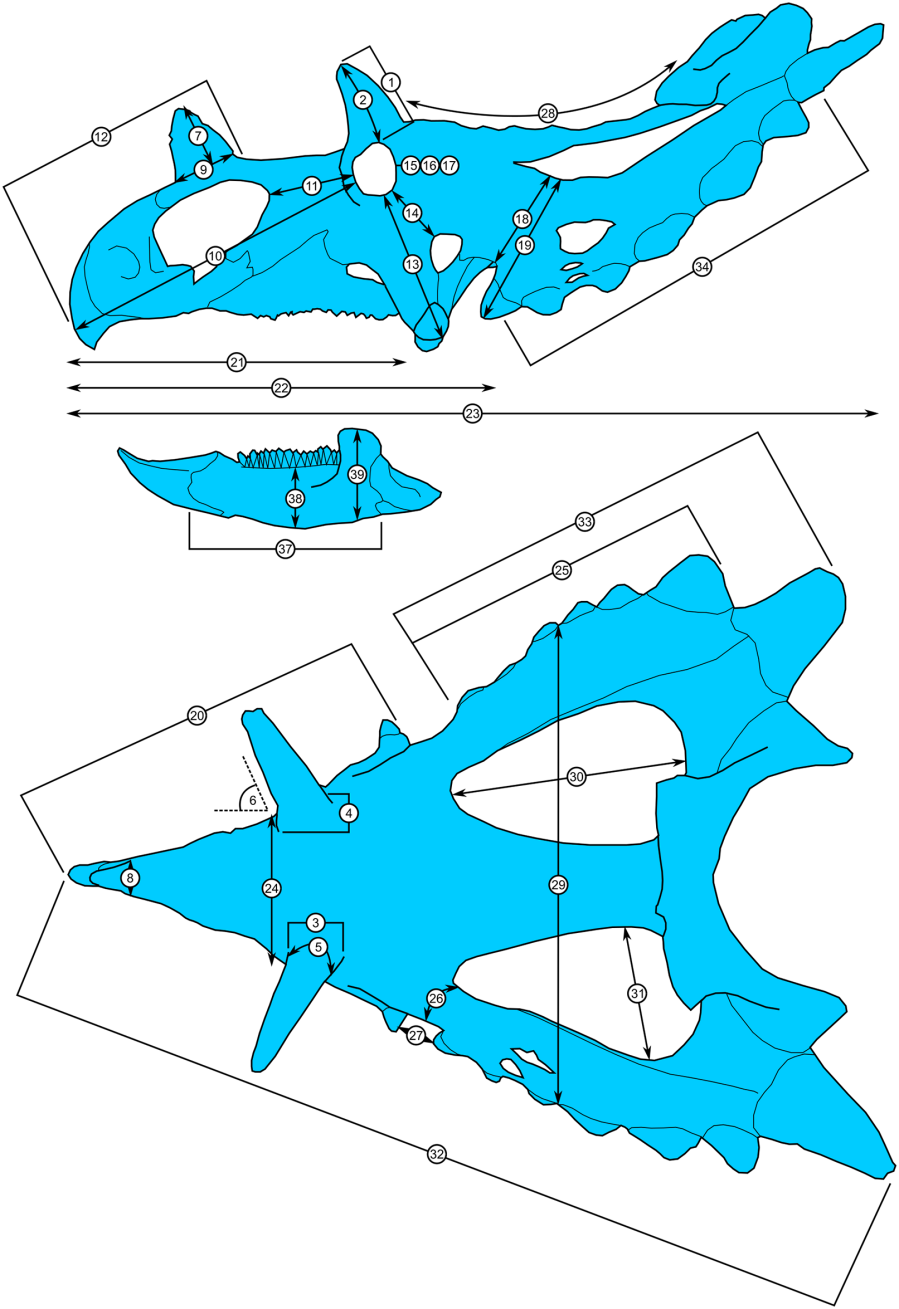
Skull measurements for *Spiclypeus shipporum* gen et sp. nov. (CMN 57081). See Table 1 for corresponding measurement descriptions and values.

The disarticulated skull is ~50% complete (Fig 3), calculated as a percentage of the total number of elements in a typical ceratopsid skull. Many of the paired bones are represented by at least one element, facilitating reconstruction by mirroring about the midline. The nasal bridge, interorbital and supratemporal regions, midline parietal bar, braincase, palate, predentary, and many of the postdentary bones are missing. The reconstructed skull is large (Table 1; Fig 4), approximating that of *Chasmosaurus* in overall size.

**Table 1:** Measurements for the holotype skull of *Spiclypeus shipporum* gen et sp. nov. (CMN 57081).

	Measurement	Value(s) (left/right)
1	Postorbital horncore length (rectilinear) from dorsal rim of orbit to apex	228/246
2	Postorbital horncore length (curvilinear) from dorsal rim of orbit to apex	231/242
3	Postorbital horncore anteroposterior length at base	102/104
4	Postorbital horncore mediolateral width at base	88/92
5	Postorbital horncore circumference about base	325/319
6	Postorbital horncore angle lateral to saggital plane in dorsal view	64°/66°
7	Nasal horncore height from base to apex (excluding nasal bridge)	166
8	Nasal horncore transverse width at base	77
9	Nasal horncore anteroposterior length at base	141
10	Rostral-orbit length	709*/691*
11	Posterior margin of external naris-orbit length	196*/181*
12	Rostral-posterior margin of nasal horncore	546*
13	Epijugal-orbit length (along surface)	333*/338*
14	Lateral temporal fenestra-orbit length	141*/174*
15	Orbit anteroposterior length	94*/84*
16	Orbit dorsoventral height	105*/96*
17	Maximum orbit diameter	107*/100*
18	Minimum distance from jugal notch to medial margin of squamosal	215*/218*
19	Minimum distance from lateral margin of anteriormost episquamosal to medial margin of squamosal	341/327
20	Rostral-epijugal (rectilinear)	867*/852*
21	Rostral-posterior edge of maxillary tooth row	735*/702*
22	Basal skull length (rostral-occipital condyle)	913*
23	Maximum anteroposterior length of skull (rostral-posterior parietal, excluding epiossifications)	1670*
24	Distance between orbits	318*
25	Length of squamosal from jugal notch to its distal end	680/720
25a	Maximum length of squamosal from apex of anteriormost episquamosal to distal end	811/736
26	Jugal notch-parietal fenestra (curvilinear)	253*/251*
27	Epijugal-squamosal (across jugal notch)	85*/134*
28	Posterior margin of postorbital horncores (approximate location of posterior margin of frontoparietal fossa) to posterior edge of medial parietal bar (curvilinear)	715*
29	Maximum transverse width of frill (at mid-length, between episquamosals)	1024
30	Maximum length of parietal fenestra	470*/459*
31	Maximum width of parietal fenestra	243*/254*
32	Maximum skull length, rostral-epiparietosquamosal	1850*/1770*
33	Jugal notch (from squamosal lateral corner) to apex of epiparietosquamosal	905/827
33a	Jugal notch (from squamosal lateral corner) to posterior margin of parietal (not including epiparietals)	771/745
34	Occipital condyle-posterior margin of frill	612*
35	Number of episquamosals	6/7
36	Number of epiparietals	6 (3/side)
37	Dentary length, from anterior margin to posterior edge of coronoid base	444/459
38	Dentary height at mid-tooth row length	?/131
39	Dentary height at coronoid process	?/214

Numbers in left column correspond to those in Fig 4. All measurements in mm unless otherwise noted. Asterisk (*) denotes reconstructed/estimated value.

#### Rostral bone

**Fig 5:**
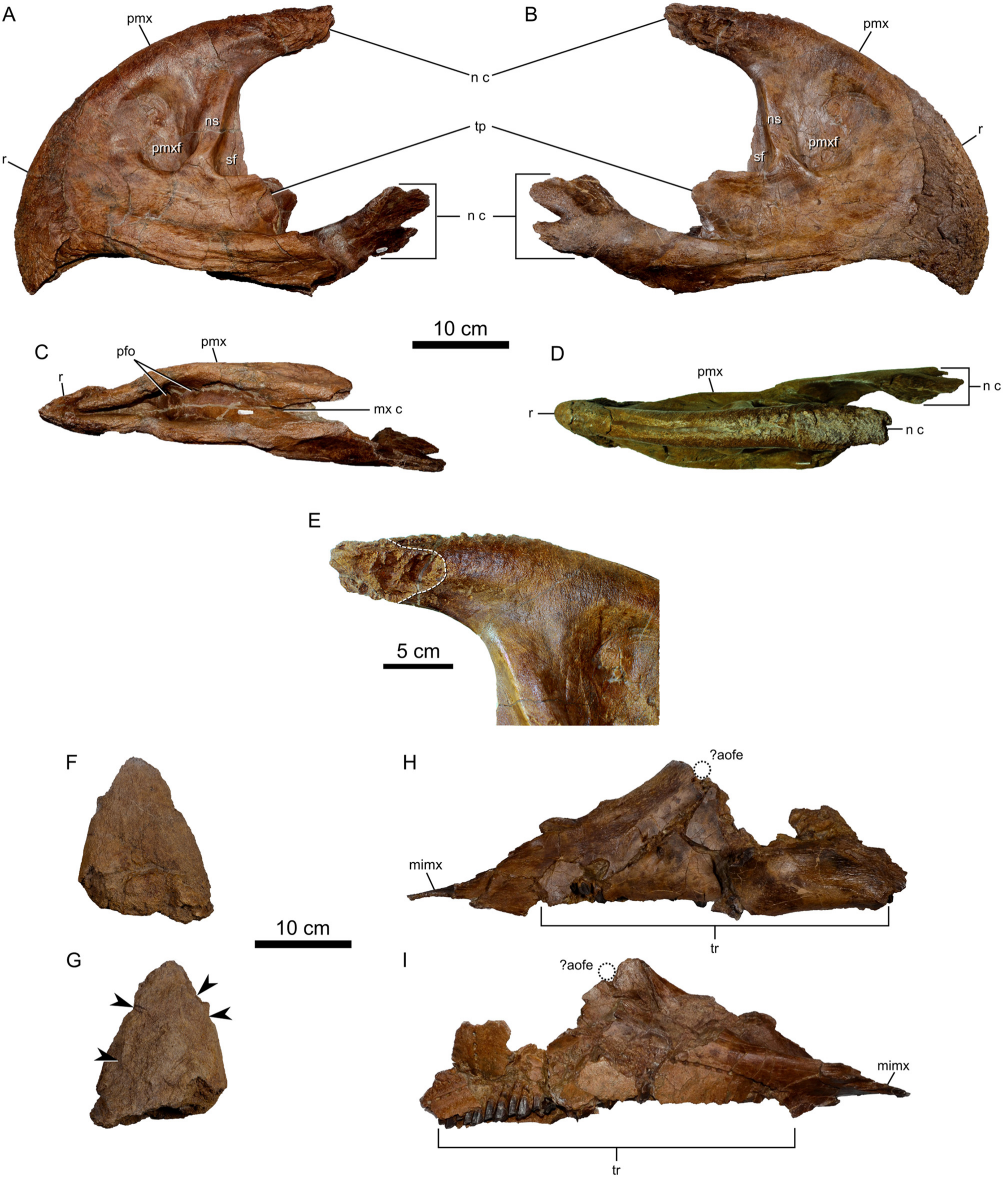
Snout elements of *Spiclypeus shipporum* gen et sp. nov. (CMN 57081). Rostral bone and premaxillae in left lateral (A), right lateral (B), ventral (C), and dorsal (D) views; (E) dorsal nasal contact of premaxilla in right lateral view, outlined by white dashed line; nasal horncore in lateral views (F and G, precise orientation difficult to discern); left maxilla in lateral (H) and medial (I) views. Arrow heads indicate resorption pits on nasal horncore.

The wedge-shaped rostral bone (Fig 5A–5D) is transversely compressed, and the left ventral ramus has broken and shifted medially. The bone is firmly attached to the underlying premaxillae, but remains suturally distinct. It is typically chasmosaurine in morphology [1], having elongate dorsal and ventral rami, subequal in length. The dorsal ramus extends posteriorly above the anterior margin of the premaxillary fossa, and the ventral rami extend posteriorly below the mid-point of the premaxillary fossa. The rostral beak is strongly hooked, with a pronounced ventral deflection. This condition is similar to that seen in *Chasmosaurus* ([42]: Figs 1, 2) and *Anchiceratops* ([25]: Fig 5), but contrasts with that of *Kosmoceratops* ([35]: Fig 5) and *Arrhinoceratops* ([36]: Fig 4) where the beak is weakly hooked. The bone surface is variably pitted and grooved, as in other ceratopsids, suggesting a horny covering in life.

#### Premaxilla

**Fig 6:**
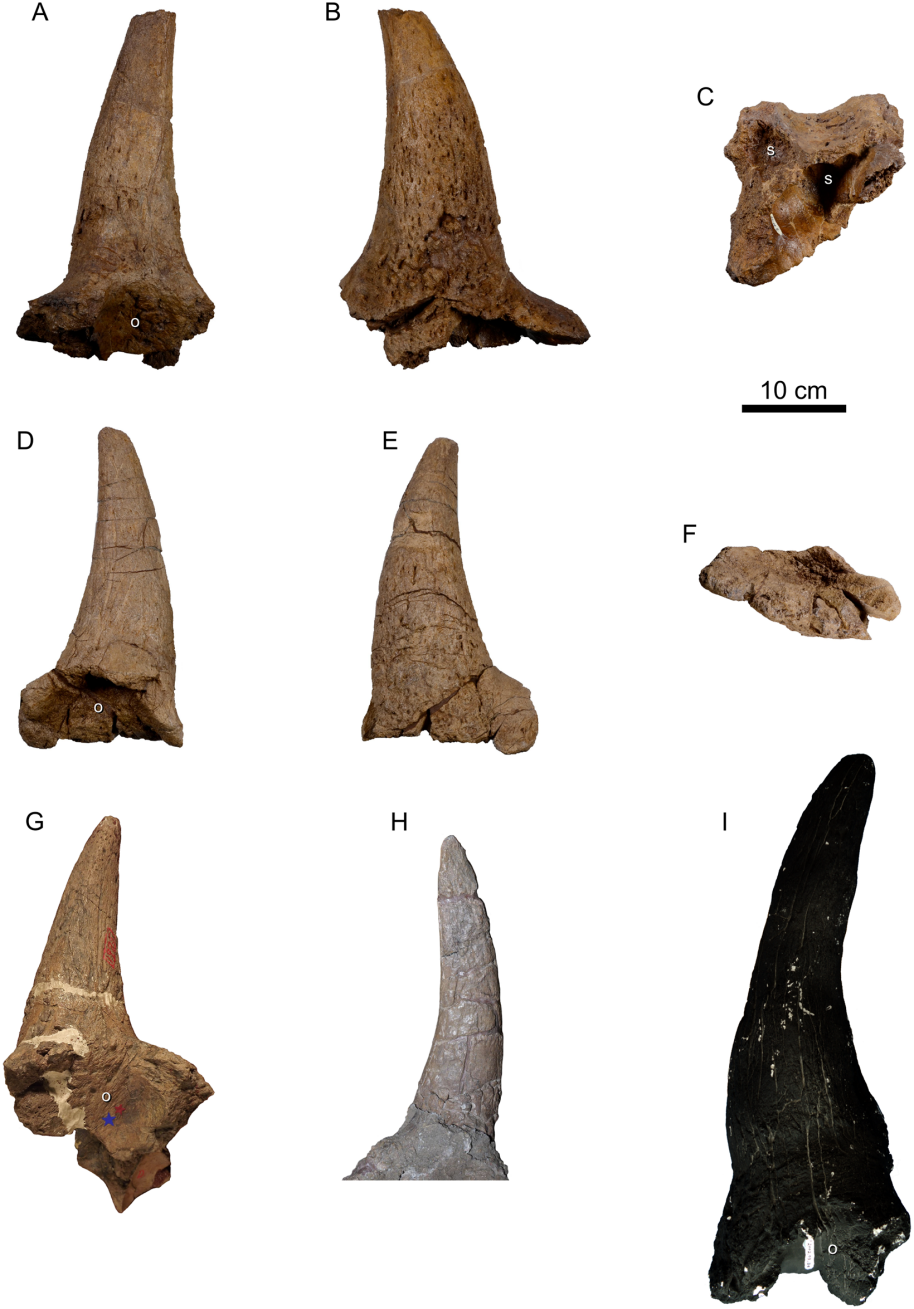
Postorbital horncores of *Spiclypeus shipporum* gen et sp. nov. (CMN 57081) and other chasmosaurines. Right postorbital horncore of *S*. *shipporum* (CMN 57081) in ventrolateral (A), dorsomedial (B), and ventral (C) views; left postorbital horncore of *S*. *shipporum* (CMN 57081) in ventrolateral (D), dorsomedial (E), and ventral (F) views; left postorbital horncore of ‘*Ceratops montanus*’ (USNM 2411) in ventrolateral view (G); left postorbital horncore of *Kosmoceratops* (UMNH VP 17000; courtesy of UMNH) in ventrolateral view (H); right postorbital horncore of *Albertaceratops* (TMP 2002.003.0036) in ventrolateral view (I).

The paired premaxillae (Fig 5A–5E) are transversely compressed, and the ventral process of the left element is missing, but they are otherwise well preserved. The premaxillae are firmly coossified about the midline, but a faint suture (now reinforced with cement) can be discerned over most of their contact. The premaxilla is longer than tall (375 mm long x 296 mm tall), as in other chasmosaurines [43]. The dorsal margin of the premaxilla is transversely thickened and rugose. The dorsal process is especially rugose laterally, bearing many deep, contiguous pits over the sutural contact for the dorsal process of the nasal (Fig 5E), which presumably reflects interdigitation of the dorsal premaxilla-nasal suture in life. This condition of the premaxilla contrasts markedly with the very smooth dorsal contact surface for the nasal in *Chasmosaurus belli* ([44]: fig 5A), and appears to be unique to CMN 57081. The restriction of the irregular surface to the hemicircular area that defines the nasal contact laterally suggests that the origin of this irregularity is neither pathological nor ornamental. The premaxillary septum is shallowly excavated laterally by a large (95 x 64 mm), oblong premaxillary fossa, which is not perforated by an interpremaxillary fenestra as it is in *Anchiceratops* ([25]; fig 6), *Utahceratops* ([35]: fig 4), and *Regaliceratops* ([34]: fig 2), even though the bone in this region is < 1 mm thick. A smaller (~20 x 20 mm) fossa occurs anterior to the larger premaxillary fossa on each of the paired elements. Posterior to the large premaxillary fossa, the robust narial strut is inclined posteriorly at an angle of 3 degrees from vertical. A thin septal flange emerges posteriorly from the narial strut for most of its height, increasing in anteroposterior length ventrally. This condition is similar to that of *Kosmoceratops* ([35]: fig 6), but contrasts with that of *Chasmosaurus* ([42]: fig 1) where the flange is more pronounced dorsally, and *Anchiceratops* ([25]: fig 6) where it is absent. A laterally concave triangular process, incomplete on both sides, emerges posteriorly from the base of the narial strut, lateral to the septal flange, as in *Utahceratops* ([35]: fig 6) and *Triceratops* ([45]: fig 1). The ventral margin of the premaxilla is transversely thickened. It bears an anteroposteriorly elongate (78 mm long on left side, 63 mm long on right side) sulcus laterally, immediately posterior to the rostral. The ventral process of the premaxilla projects posteriorly, twisting gently along its length as it curves laterally and then medially. The ventral process terminates in two posteriorly directed projections, the dorsalmost being distinctive in both its larger size and in possessing a stepped dorsal margin. A roughened facet excavates the anterodorsal surface of the dorsal projection, and would have underlapped the ventral process of the nasal when in articulation. The palatal surface of each premaxilla bears two parasagittally aligned fossae, of which the posteriormost aligns with the posterior edge of the rostral bone. The premaxillae diverge from the midline further posteriorly to receive the medially inflected anterior processes of the maxillae (see [46]: fig 26).

#### Nasal horncore

The nasal horncore (Fig 5F and 5G) is preserved in isolation, apart from the remaining nasals, which are mostly missing (a portion of the posterior rim of the naris is preserved, but is otherwise uninformative). This situation makes the precise placement of the horncore along the nasal bridge difficult to discern. It is reconstructed here as centred over the external naris (Fig 3A and 3B), as in *Anchiceratops* ([25]: fig 5), but it is also possible that the horncore occurred over the posterior margin of the external naris, as in *Chasmosaurus* ([42]: fig 1) and *Utahceratops* ([35]: fig 6).

The horncore resembles an acute isosceles triangle in lateral view. It is modest in size (Table 1), comparing favourably with the relative proportions seen in *Chasmosaurus* ([42]: fig 1). The horncore is oval in cross-section, with the long axis oriented anteroposteriorly, as in *Chasmosaurus* ([42]: fig 1). This condition contrasts with that of *Arrhinoceratops* ([36]: fig 5), in which the anterior face of the horncore is flat, and *Anchiceratops* [25] and *Regaliceratops* [34], in which the horncore is teardrop shaped in cross-section. The symmetry of the isolated horncore about the transverse axis obscures its precise orientation. A bony rim at one end of the horncore base likely served to anchor a keratinous sheath in life. A similar bony rim is often most pronounced anteriorly in ceratopsids (e.g., *Styracosaurus albertensis*, CMN 344; *Chasmosaurus russelli*, CMN 8800), but the incompleteness of the nasal renders a precise determination impossible. The external surface of the horncore is poorly preserved; much of the internal trabecular bone is exposed. Nonetheless, several proximodistally trending vascular sulci are visible along the surface. The horncore is scored by four shallow pits, varying between 12 x 12 mm and 41 x 20 mm in surface area (Fig 5G). The origin of the pits is unclear, but they may represent ‘resorption pits’ (sensu Tanke and Farke [47]), which occur more frequently on postorbital horncores. There is no indication of an epinasal.

#### Maxilla

The left maxilla is preserved in two pieces. As restored (Fig 5H and 5I), it is approximately triangular in lateral outline and measures 494 mm along the ventral margin. The medially inset tooth row (336 mm long) extends most of the ventral length of the maxilla, and although poorly preserved in places, a minimum of 28 tooth positions can be discerned. Anterior to the tooth row, the narrow edentulous portion of the maxilla is laterally exposed for 63 mm. The anterior face of the maxilla slopes posterodorsally at an angle of 29 degrees relative to the tooth row. A short (26 mm long) medially extending shelf occurs midway up the length of the anterior face, and likely would have braced the overlying premaxilla-nasal contact. The apex of the maxillary ascending process bears a posteriorly facing groove to receive the lacrimal ([46]: fig 21), and is situated 141 mm dorsal to the midpoint of the tooth row. A subtle notch is visible laterally, immediately posterior to the apex of the ascending process, and may represent the inferior margin of the highly reduced antorbital fenestra, but the bone is too incomplete here to be certain. The posterior face of the maxilla is badly damaged, precluding description of the contacts for the jugal and pterygoid. The maxilla tapers posteriorly to form a tooth-bearing posterior process. The external face of this process is dorsomedially-ventrolaterally inclined, and a roughened surface at the posterior end of the process marks the likely contact for the missing ectopterygoid ([42]: fig 2).

The lateral surface of the maxilla is generally flat, with little evidence for the pronounced maxillary ridge seen, for example, in *Chasmosaurus* ([44]: fig 2), although its absence may have resulted from damage to the specimen. The bone surface is smooth, though marked in places by several small foramina, particularly along the anterior border. A single large (12 x 8 mm) foramen occurs near the centre of the maxilla, below the apex of the ascending process.

The medial surface of the maxilla is poorly preserved, although the supradental plate covers most of the tooth battery. A series of special foramina (sensu Edmund [48]), whose numbers approximately correspond to the number of tooth positions, runs the length of the plate along the base of the tooth battery. Anterior to the tooth row, the maxilla inflects medially to form a thin secondary palate that would have slotted into the V-shaped trough formed anteriorly by the premaxillae. Here, the medial contact for the adjoining premaxilla is longitudinally fluted. The medial inflection of the maxilla forms a trough that runs the along the anterior face of the element, and that would have supported the overlying ventral process of the premaxilla.

#### Postorbital

Both postorbitals are preserved with their respective horncores (Fig 6A–6F). The right element is missing the horncore tip (preserved on the left side), but is otherwise more complete and less dorsoventrally crushed than its counterpart. The dorsal rim of the orbit is 77 mm across, as preserved. The internal wall of the orbit is smooth but punctured in places by several small (2–10 mm diameter) foramina. The medioventral surface of the postorbital is invaded by two diverticulae of the supracranial sinus system [49]. The smaller one (16 cm^2^, 1.5 cm deep) is located immediately posterior to the orbit, and the larger cornual diverticulum (46 cm^2^, 4 cm deep) occurs medial to the orbit but does not invade the base of the horncore as in *Pentaceratops* and *Triceratops* ([50]: fig 1).

The robust postorbital horncore is centred dorsal to the orbit. The horncore is oval in cross-section and is comparable in length to that of ‘*Ceratops*’ and *Kosmoceratops* (Table 1; fig 6G and 6H). It projects dorsolaterally above the orbit and gradually curves ventrolaterally towards the distal end. The strong lateral projection of the horncore (50 degrees from vertical in anterior view) is rare among ceratopsids, and is otherwise seen only in *Albertaceratops* [5], *Utahceratops*, and *Kosmoceratops* [35]. There is only the subtlest indication of an anterior curvature to the horncore when viewed laterally (Fig 3A and 3B). The horncore is rugose and heavily pitted near its base, but these features give rise to apicobasally elongate vascular sulci midway up the length of the horncore.

#### Jugal

**Fig 7:**
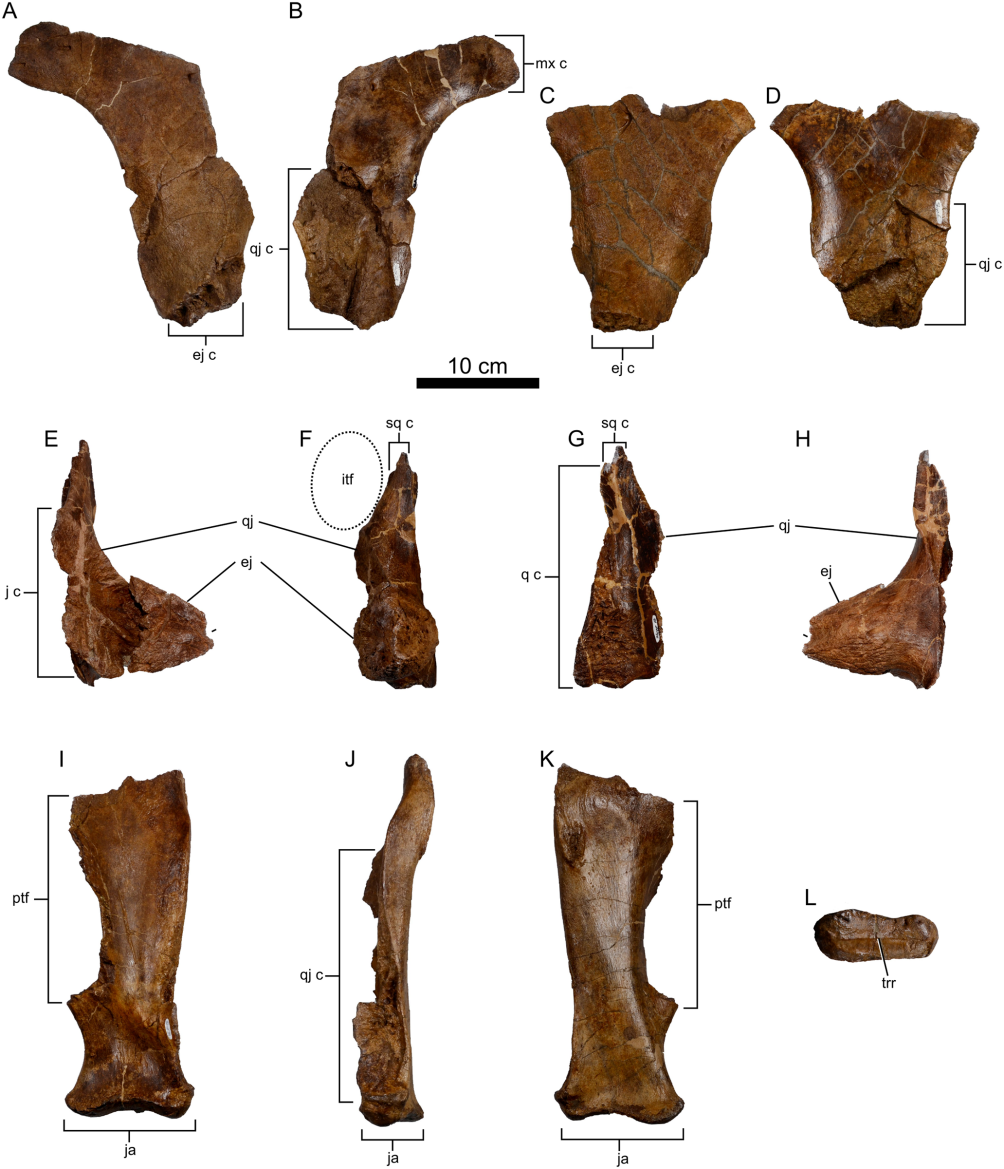
Infratemporal elements of *Spiclypeus shipporum* gen et sp. nov. (CMN 57081). Left jugal in lateral (A) and medial (B) views; right jugal in lateral (C) and medial (D) views; left quadratojugal and epijugal in anterior (E), lateral (F), medial (G), and posterior (H) views; left quadrate in anterior (I), lateral (J), posterior (K), and ventral (L) views.

Two partial, flat jugals are preserved (Fig 7A–7D). The left element retains a portion of the anterior process, which bears a shallow facet medially to overlap the ascending process of the maxilla. The facet is incomplete where it abuts the dorsally broken edge of the jugal but, as preserved, it is D-shaped in outline. There is a small (2 x 2 mm) blind foramen in the posterodorsal corner of the facet.

The ventral process of the jugal is V-shaped in lateral profile, with gently sinusoidal anterior and posterior margins, as in other ceratopsids. The process thickens from 15 mm dorsally to 32 mm ventrally. Here, the apex of the ventral process flares slightly laterally, and bears an elongate and highly rugose scar that trends across the lateral surface in an anteroventral-posterodorsal direction. This marks the attachment site of the epijugal. There is no posteriorly directed infratemporal process as in *Chasmosaurus* ([42]: fig 1). Medially, the ventral half of the ventral process is excavated by a shallow, rugose facet for the underlapping quadratojugal. The margin of the facet traces a gradual arc across the medial surface of the left jugal, from the anteroventral corner to approximately half-way up the ventral process. On the right element, the facet is more nearly square in outline. The rugose quadratojugal contact bears a deep, dorsoventrally trending channel that corresponds to a prominent ridge on the jugal contact of the quadratojugal.

The lateral face of the jugal bears a rough, woven bone texture, scoured by occasional faint vascular sulci that trend dorsoventrally. This contrasts with the smooth texture on the medial surface of the element. Both lateral and medial surfaces are punctured by occasional foramina, particularly toward the dorsal end.

#### Epijugal

Only the left epijugal is preserved (Fig 7E, 7F and 7H). It is 78 mm tall and 60 mm long at the base, and projects 75 mm laterally. Its overall morphology compares favourably with that of *Kosmoceratops* [35] or *Arrhinoceratops* [36], and contrasts with the smaller, less pronounced epijugal of *Chasmosaurus* [42, 44]. The epijugal is generally conical in shape, but with a flattened anterodorsal face. The external surface bears a woven bone texture scored by numerous pits and apicobasally oriented vascular sulci. A 6 mm deep groove incises the posterior surface of the epijugal near the apex. The floor of the groove is heavily pitted and lacks the vascular sulci seen over the remaining surface of the bone. The groove is consistent with instances of cranial pitting reported in centrosaurine epijugals [47, 51]. Although the epijugal caps both the jugal and quadratojugal, it is only fused to the latter. The suture between these elements is poorly delineated; only the sudden textural shift from the rugose epijugal to the comparably smooth quadratojugal betrays the contact externally.

#### Quadratojugal

The left quadratojugal is nearly entirely preserved (Fig 7E–7H); only a portion of the jugal contact is missing. The pyramidal element is dorsoventrally tall (198 mm), pinching out between the squamosal and quadrate dorsally. The external surface of the quadratojugal twists through an arc of ~130 degrees about the long axis, transitioning from a posteriorly facing orientation ventrally to a laterally facing orientation dorsally. It generally bears a smooth, long-grained texture, except near the contact for the epijugal, where the surface texture is mottled (sensu Brown et al. [52]). The broad contact for the overlapping jugal faces anterolaterally, and extends two-thirds of the way up the length of the quadratojugal. The contact surface is heavily striated, and a prominent ridge extends from the epijugal contact across the articular surface for the jugal in a dorsoventral orientation. This ridge slots into a corresponding groove on the quadratojugal contact surface of the jugal, imparting great strength to this complex. The contact surface for the quadrate twists through an arc of 90 degrees, so that it transitions from a medially facing orientation ventrally to a posteriorly facing orientation dorsally. The contact is extremely rugose ventrally and smooth dorsally. The medial trough between the quadrate and jugal contact surfaces is smooth.

#### Quadrate

Both quadrates are partially preserved, the left (Fig 7I–7L) being much more complete than the right. As preserved, the left quadrate is 277 mm tall, measured along the lateral surface. The quadrate is anteroposteriorly narrow, and mediolaterally wide. The main body of the element twists through an arc of ~45 degrees, so that its external surface transitions from a posterior orientation ventrally to a posteromedial orientation dorsally. The medial pterygoid flange is mostly missing; the little that remains indicates that it would have extended two-thirds of the way down the height of the quadrate, as in other ceratopsids [46]. Ventrally, the quadrate expands into two transversely arrayed condyles for articulation with the lower jaw. The medial condyle occupies approximately two-thirds of the articular surface, which measures 100 mm wide, and a very low bony ridge transversely extends across the surface (Fig 7L). The highly rugose contact for the quadrojugal is offset from the lateral condyle by ~20 mm, is teardrop shaped in outline, and extends two-thirds of the way up the lateral surface of the quadrate. Anteriorly, the quadrate is shallowly excavated immediately dorsal to the condyles; this fossa possibly served as the attachment site for the m. adductor mandibulae posterior in life [53, 54]. A rugose, elliptical scar (25 mm long x 19 mm wide) occurs on the posterolateral surface of the quadrate, and possibly marks a point of articulation for the paraoccipital process of the occiput.

#### Parietal

**Fig 8:**
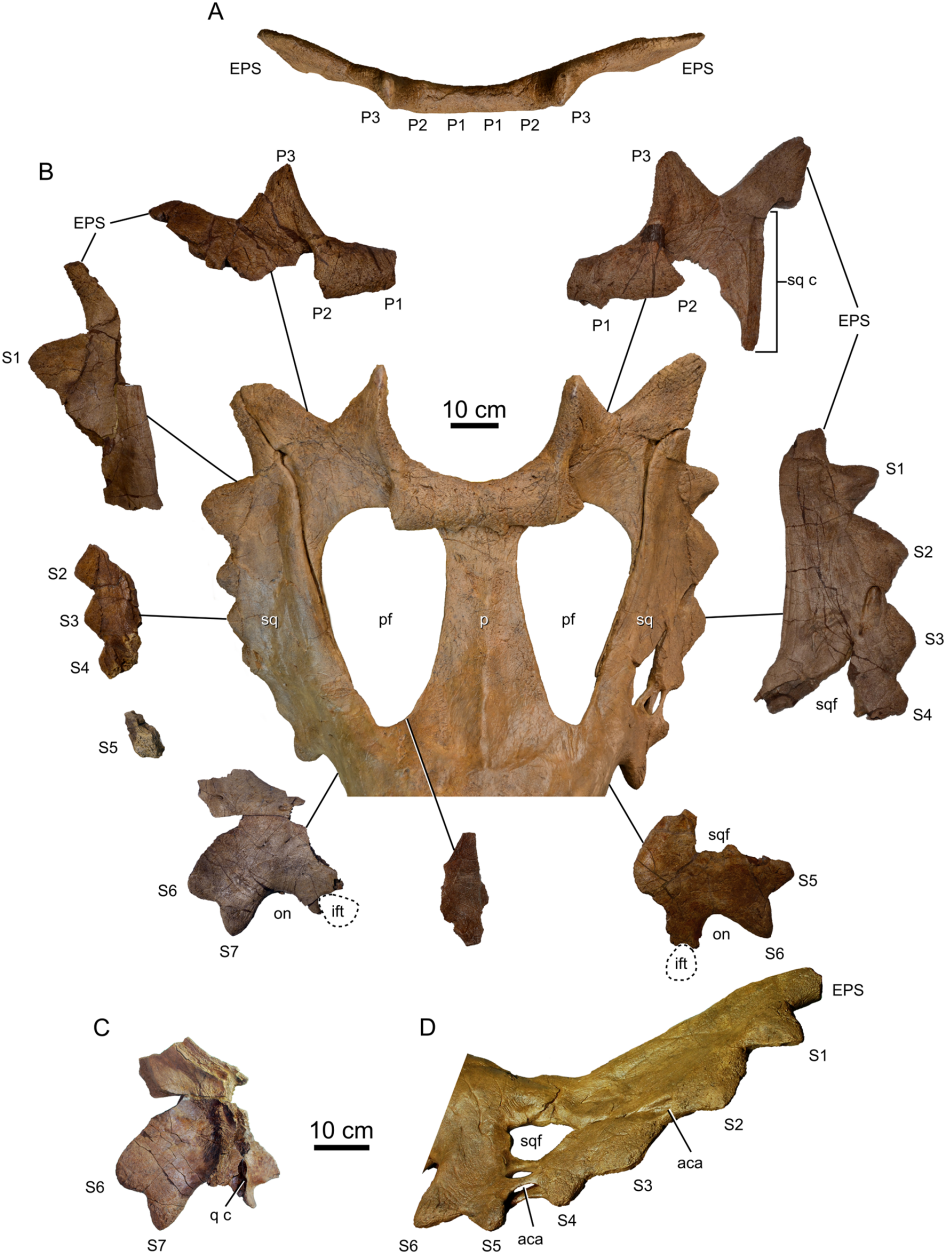
Parietosquamosal frill of *Spiclypeus shipporum* gen et sp. nov. (CMN 57081). (A) Posterior parietal bar in posterior view; (B) reconstructed frill and related fossil material; (C) portion of anterior right squamosal in medial view; (D) reconstructed left squamosal showing fenestrae and associated abscess cavities.

**Fig 9:**
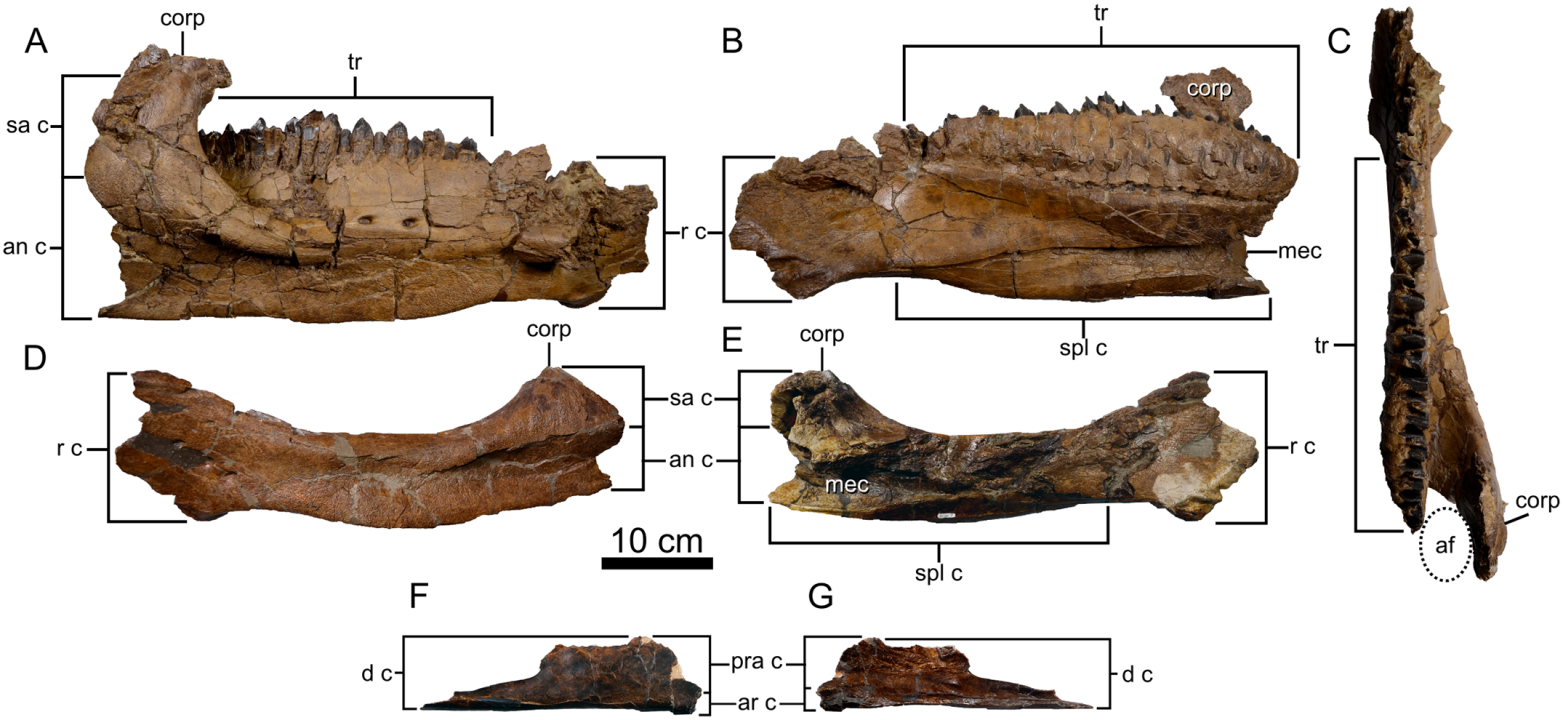
Lower jaw elements of *Spiclypeus shipporum* gen et sp. nov. (CMN 57081). Right dentary in lateral (A), medial (B), and dorsal (C) views; left dentary in lateral (D) and medial (E) views; right splenial in medial (F) and lateral (G) views.

The parietal (Fig 8A and 8B) is missing most of the median and lateral bars. The posterior parietal bar is anteroposteriorly broad, as in *Pentaceratops* [55] and *Utahceratops* [35], and quite unlike the strap-like condition seen in *Chasmosaurus* [42, 44]. The exact dimensions are difficult to discern, owing to missing bone around most of the parietal fenestrae, but based on the little marginal bone that remains, the posterior bar appears to have varied in anteroposterior breadth between 195 mm laterally and 115 mm medially. The posterior bar is ~40 mm thick between the adjoining epiparietals, and thins anteriorly to just 3 mm around of the posterior margins of the parietal fenestrae. A shallow sulcus arcs across the dorsal surface of the posterior bar on each side, roughly paralleling the posterior margin of the parietal fenestra, before disappearing beneath the forward curling epiparietals. The posterior bar bears a broad medial embayment, comparable to that of *Chasmosaurus russelli*, *Mojoceratops* ([56]: fig 9), and *Kosmoceratops* ([35]: fig 6), and is not so strongly embayed as in either *Pentaceratops* ([55]: fig 6) or *Utahceratops* ([35]: fig 4). From the posteriormost point of the parietal (between epiparietal 3 and the epiparietosquamosal), the embayment is 120 mm deep.

The parietal rapidly tapers laterally to form strap-like lateral bars, best preserved on the left side. Here, the lateral bar is widest (48 mm) along its roughened, dorsolaterally facing contact for the squamosal. The lateral bar is just 14 mm thick perpendicular to this contact. Although broken at mid-length, the corresponding parietal facet on the squamosal indicates that the lateral bar was continuous and uniformly wide before expanding ventrally to adjoin the midline bar in a broad parietal apron near the base of the frill. A portion of this apron is preserved where it forms the anteromedial rim of the right parietal fenestra. The fragment is dorsally rugose and covered in a network of vascular sulci on both its dorsal and ventral surfaces. A small (20 mm long x 14 mm wide x 11 mm deep) and internally smooth punched out lesion (sensu Tanke and Farke [47]) occurs in the thickest (21 mm) portion of the fragment near the base of the frill.

Given the incompleteness of the parietal, the precise dimensions of the parietal fenestrae are uncertain, particularly along their border with the unpreserved median bar. However, from the few marginal sections that remain, it is clear that the fenestrae occupied a large portion of the frill, laterally abutting the squamosals, and were anteroposteriorly elongate, perhaps measuring up to 460 mm long, as in our reconstruction (Fig 8B). We have reconstructed the frill with a broad median parietal bar, as in *Anchiceratops* [25] and *Arrhinoceratops* [36], producing a maximum fenestral width of 260 mm; however, if the median bar was transversely narrower, as in *Chasmosaurus* [42, 44], each fenestra may have been up to 50 mm wider.

#### Squamosal

Portions of both squamosals are preserved (Fig 8B–8D), with the left being more complete. The squamosal resembles a scalene triangle, is elongate, and gradually tapers posteriorly, as in other chasmosaurines [43]. The anterior butt joint for the postorbital is broad and sinusoidal, extending from the lateral to the dorsal surface of the skull. A short infratemporal process projects ventrally from the anterior squamosal to form the posterodorsal margin of the laterotemporal fenestra. The complex formed by the squamosal, quadratojugal, and jugal indicates that the enclosed infratemporal fenestra was smaller than the orbit, as in *Chasmosaurus* ([42]: fig 1) and *Pentaceratops* ([55]: fig 6), and not subequal in size to the orbit, as in *Arrhinoceratops* ([36]: fig 4) and *Utahceratops* ([35]: fig 4). The quadratojugal ascends the posterior margin of the squamosal infratemporal process to insert into a small fossa on the ventral surface of the squamosal. Immediately posterior to this fossa, a deep groove excavates the medial surface of the squamosal to receive the quadrate (Fig 8C), and posteriorly adjacent to this, a gently concave thickening of the bone served to brace the paroccipital process of the occiput.

The posterior ‘blade’ of the squamosal is weakly convex laterally; the transversely widest point of the frill would have occurred at mid-length. The undulating lateral margin is up to 45 mm thick between the episquamosals. The squamosal blade thins centrally to form a dorsal concavity, and thickens once again towards the medial contact with the parietal. Here, the medioventral surface of the blade is excavated by a rugose, shallow facet for the parietal. The posterior end of the squamosal nearly extends to the posterior corner of the frill and terminates in a blunt point, as in *Anchiceratops* ([57]: fig 2). Both the dorsal and ventral surfaces of the squamosal blade are rugosely textured, and faint vascular traces run longitudinally across the surface.

The dorsal surface of the right squamosal is incised by three small (≤ 15 x 10 mm), smooth-walled punched out lesions (sensu Tanke and Farke [47]) near the jugal (otic) notch. The left squamosal blade is more extensively remodelled where it is perforated by three large and closely spaced fenestrae (Fig 8D). The largest (159 x 80 mm) and medialmost fenestra resembles a puckered lesion (sensu Tanke and Farke [47]) in being recessed and surrounded by up to three radiating fissures. A second (60 x 34 mm) fenestra occurs lateral to the first, and a third (79 x 19 mm) opens laterally where it has eroded into the bordering episquamosals S4 and S5. The three fenestrae are separated from one another by three smooth bony struts, which are missing but can be inferred from their broken bases. A smooth trough occurs on the dorsal surface of the squamosal between episquamosals S2 and S3, and opens into a large (20 x 20 mm), blind foramen.

Although unilateral fenestrae are common in the squamosals of chasmosaurines, they are said to never occur near the external margins, and are thought to form through normal processes due to the usual lack of any signs of trauma or disease [47]. However, CMN 57081 is unique in having multiple squamosal fenestrae, one of which occurs on the external margin, and in that the various openings are associated with what are reasonably interpreted as drainage (fistulous) tracts. As such, the squamosal fenestrae of CMN 57081 are apparently pathological in origin, and may represent the effects of chronic osteomyelitis [58].

#### Frill epiossifications

The frill epiossifications (Fig 8) are among the most distinctive features of the skull, and are described here using the standardized terminology of Sampson et al. [35]. All are variably pitted and scoured by vascular sulci externally. Three triangular epiparietals occur on either side of the parietal. All epiparietals are indistinguishably fused at their bases, as in *Vagaceratops* [59] and *Kosmoceratops* [35], yet remain suturally distinct from the parietal. Epiparietals P1 and P2 emerge from the dorsal surface of the posterior parietal bar and curl anteriorly so that the apex of P1 points anteriorly over the parietal fenestra and the apex of P2 points anterolaterally. Curled epiparietals also occur in *Chasmosaurus*, *Mojoceratops*, and *Utahceratops*, but in these forms the apices of the epiparietals point dorsally. In CMN 57081, the anterior curvature of epiparietals P1 and P2 more closely resembles that of *Vagaceratops* and *Kosmoceratops*. However, unlike in these last two forms, epiparietals P1 and P2 are so thoroughly fused to each other that they are nearly indistinguishable except for their apices. The base of P2 flourishes laterally to join the twisted and posterodorsally directed P3.

A large epiparietosquamosal (EPS), subequal in size to P3 (Table 2), caps the parietal-squamosal suture. Unlike P3, whose medial edge laps onto the dorsal surface of the parietal, the EPS lies entirely within the plane of the frill and the apex points posterolaterally. On each side, the EPS is split where it overlies the parietal-squamosal suture.

**Table 2:** Epiossification measurements (in mm) for the holotype of *Spiclypeus shipporum* gen et sp. nov. (CMN 57081).

	Left		Right	
Position	Base-apex	Basal width	Base-apex	Basal width
P1	112	67	115	81
P2	136	140	145	150
P3	213	190	150	172
EPS	171	200	200	195
S1	109	161	131	114
S2	62	102	109	173
S3	50	100	68	142
S4	25	84	41	69
S5	35	79	34	76
S6	32	70	64	83
S7	68	76	--	--

There are six episquamosals on the left side and seven on the right (although the reconstruction does not incorporate the isolated right S5 episquamosal and instead shows six; Fig 8B). The anteriormost episquamosal resembles an equilateral triangle, is modestly sized (Table 2), and points anteriorly into the jugal (otic) notch. Moving posteriorly, the following four or five episquamosals are obtuse and become progressively larger in size (Table 2). The posteriormost episquamosal is acute and intermediate in height between the adjacent epiossifications.

#### Dentary

Both dentaries are preserved (Fig 9A–9E), but the right is more complete than the left. The tooth row (318 mm long) extends most of the length of the dentary, and although poorly preserved anteriorly, there is room to accommodate no less than 24 tooth positions. The edentulous anterior end of the dentary is slightly inflected medioventrally where it would have been capped by the missing predentary. An elongate and depressed facet occurs anteroventrally on the lateral surface of the dentary to underlap the lateral inferior process of the predentary. Below this, a bony lip projects medioventrally to receive the medial inferior process of the predentary along a vertical lap joint. The ventral margin of the dentary is rugose and weakly convex ventrally. Posteriorly, an enlarged coronoid process emerges from the lateral surface of the dentary and projects vertically 55 mm above the medially inset tooth row. There is no pronounced lateral dentary flange extending anteriorly from the base of the coronoid process as in *Anchiceratops* [25] and *Arrhinoceratops* [36], although medial crushing of the right element gives this impression. The apex of the coronoid process is sagittally expanded. The posterior expansion of the coronoid process is slightly depressed and roughened where the external adductor musculature presumably attached in life [60]. The lateral surface of the dentary is perforated by several large (11 x 6 mm) foramina near the centre of the element and further anteriorly near the predentary articulation.

Medially, the adductor fossa opens between the coronoid process and the posterior end of the tooth. The fossa opens ventrally into the medially exposed Meckelian canal, which progressively shallows until it terminates beneath the anterior end of the tooth row. The bone surface above the posterior end of the canal is fluted where the splenial would have overlapped. Further dorsally, the supradental wall is intact, obscuring the underlying dentition. As in the maxilla, the base of the medial wall is perforated by an anterodorsally trending series of special foramina (sensu Edmund [48]), whose numbers approximately correspond to the number of tooth positions.

#### Splenial

The right splenial (Fig 9F and 9G) is partially preserved. It is cleaver-shaped in outline, with a dorsal expansion posteriorly. The splenial inflects medially in advance of the expansion to underlap the dentary. Based on the size of the corresponding facet on the dentary, ~70 mm are missing from the anterior end of the splenial.

#### Dentition

**Fig 10:**
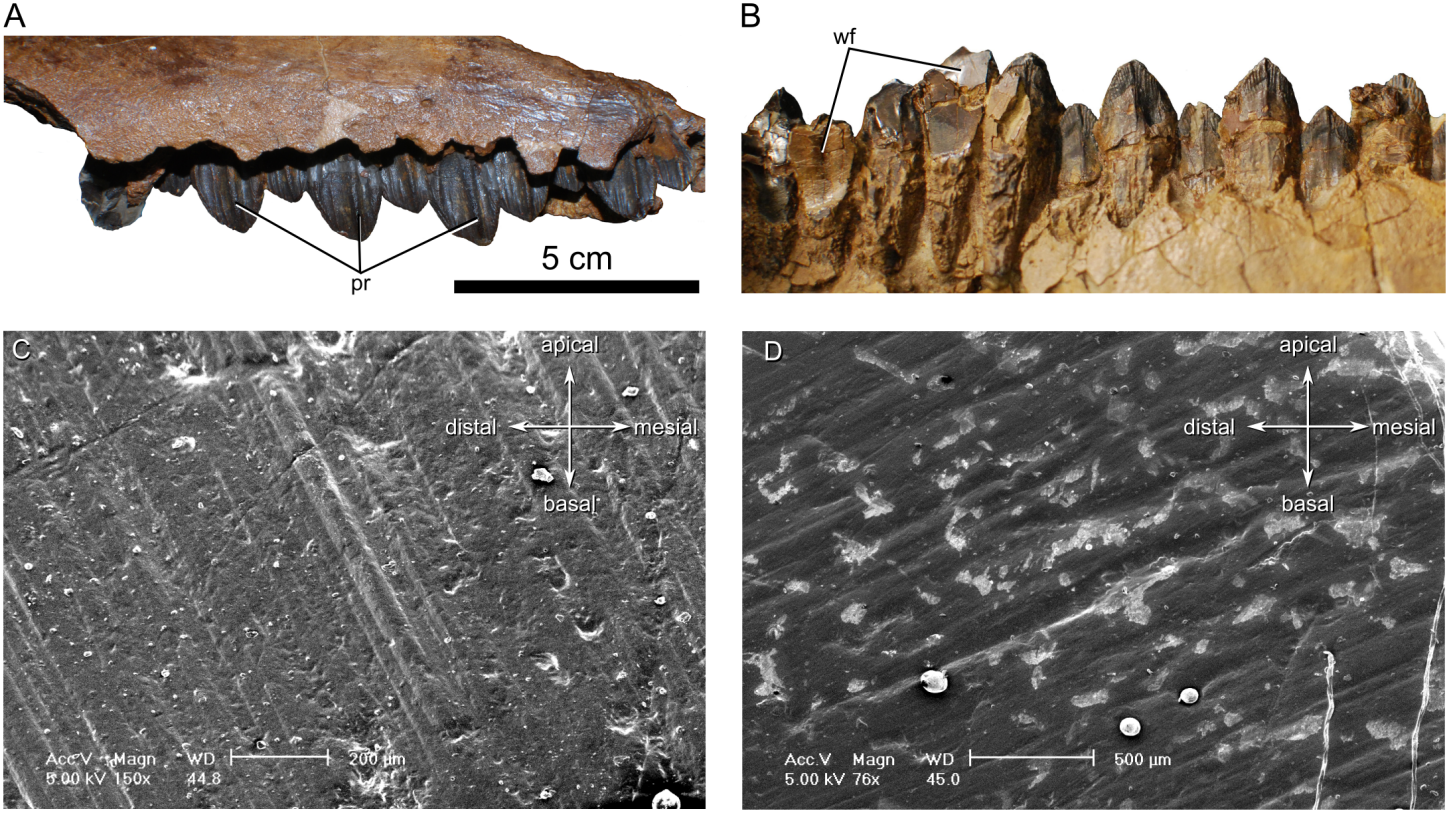
Teeth of *Spiclypeus shipporum* gen et sp. nov. (CMN 57081). (A) Left maxillary teeth in lateral view; (B) right dentary teeth in lateral view. (C) microwear on the 23^rd^ right dentary tooth showing common dorsodistally-ventromesially oriented scratches; (D) microwear on the 13^th^ right dentary tooth showing less common dorsomesially-ventrodistally oriented scratches.

Teeth are preserved in the left maxilla (Fig 10A) and right dentary (Fig 10B), but not in the left dentary. They closely resemble those of other ceratopsids [61] in that each tooth has a lanceolate crown capped by enamel on only one side, a vertical wear facet (when present), a pronounced primary ridge opposite the occlusal surface, and one or two secondary ridges on either side of the primary ridge. The bifurcate roots typical of ceratopsids are visible on some isolated teeth. Where the supradental walls of the jaws have broken away, the tooth roots are visibly enveloped by a rough layer of cementum.

The dentary teeth can be distinguished from those of the maxilla in several respects. First, the vertical wear facets of the dentary teeth face laterally, whereas those of the maxillary teeth face medially. Second, the primary ridge of the dentary teeth is much more prominent and anteriorly offset, whereas it is posteriorly offset in the maxillary teeth. Third, although subtle fluting occurs at the apex of each tooth, continuous with the small denticles that emerge from the crown margin, the fluting is more extensive on the occlusal face of unworn dentary teeth than unworn maxillary teeth.

The only worn teeth occur in the dentary. Closer inspection of their shearing facets using scanning electron microscopy reveals dorsodistally-ventromesially oriented scratches (Fig 10C), consistent with the presumed direction of pull of the external adductor musculature derived from the power stroke [60]. Less common dorsomesially-ventrodistally oriented scratches (Fig 10D) correspond to the presumed direction of pull of the pterygoideus musculature [60].

The teeth are arranged in dental batteries in both the upper and lower jaws; however, the generally intact supradental plates make it difficult to comment on their arrangement. Where a portion of the plate has broken away from the posterior process of the maxilla, three teeth per tooth family can be discerned. There are possibly more teeth per tooth family in the taller central part of the maxilla. The supradental plate likewise obfuscates the dentary tooth battery, but where the primary ridges of the underlying teeth occasionally perforate the thin bony wall, up to four or possibly five teeth per tooth family can be seen near the middle of the dental battery.

#### Comments on the postcranium

Approximately 6% of the postcranium, calculated as a percentage of the inferred total number of elements, is preserved; however, representative elements occur throughout the postcranium. The bones are in poor condition, having suffered from post-burial deformation and weathering. The postcrania were the first elements to be found weathering out of the hillside when the specimen was discovered (Fig 1C). The preserved postcranium is morphologically conservative, differing little from that of most other ceratopsids [1].

#### Vertebrae

**Fig 11:**
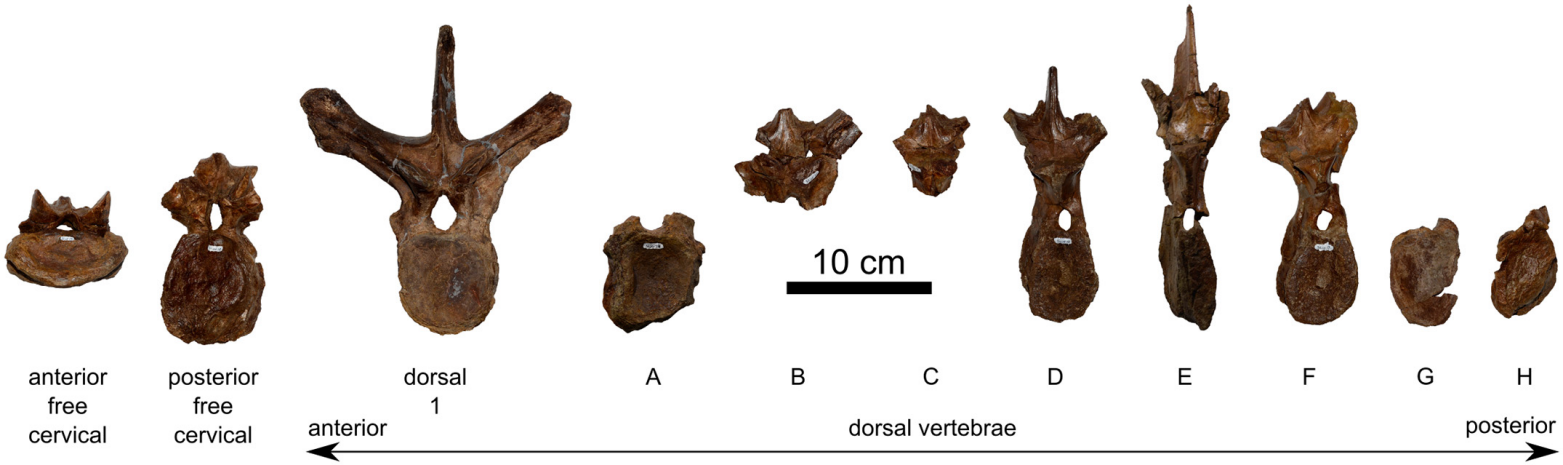
Vertebrae of *Spiclypeus shipporum* gen et sp. nov. (CMN 57081) in anterior view. Vertebrae A–H are dorsals of indeterminate position.

Several cervical and dorsal vertebrae are preserved (Fig 11), albeit poorly, having suffered from post-burial breakage and compression. Many vertebrae are transversely sheared. The poor preservation of the vertebrae prevents meaningful description of their original condition, but we detail the more salient features where possible. The more complete vertebrae show total fusion of the neural arch to the centrum, such that the sutures are externally obliterated. The incomplete preservation of the spinal column makes it impossible to ascertain the vertebral formula; however, there is no evidence to suggest that the formula deviates from the general ceratopsid condition [1].

Two free cervical vertebrae are identified by the presence of parapophyses on the centrum. Both cervicals are missing their neural and transverse spines. One element is strongly compressed dorsoventrally, hindering its precise identification. It is tentatively identified as an anterior free cervical (C4, C5, or C6), based on the enlarged centrum (Table 3) and its vaguely heart shaped anterior and posterior faces, and the anteroposterior exposure of the zygapophyseal facets [62]. A pair of parasagittal ridges occurs on the ventral surface of the centrum, which contrasts with the single median ridge borne by centra C4–C6 of *Chasmosaurus* [45]. A second element is interpreted as a posterior cervical (probably C7 or C8), based on the less anteroposteriorly elongate centrum (Table 3) and its more rounded anterior and posterior faces. There is no median keel immediately beneath the postzygapophyses, as occurs in vertebra C9 of *Styracosaurus* [62] and *Triceratops* [63]. On both preserved cervicals, a faint midline keel, likely for the attachment of the interspinous ligament [63], occurs immediately above the prezygapophyses and ascends the base of the neural spine.

**Table 3:** Vertebral measurements for *Spiclypeus shipporum* gen et sp. nov. (CMN 57081).

ID	Centrum length (mm)	Centrum height (mm)	Centrum width (mm)	Neural canal height (mm)	Neural canal width (mm)	Angle between prezygapophyses (°)	Angle between postzygapophyses (°)	Angle between transverse processes (°)	Angle of neural spine from vertical (°)
anterior free cervical	74	68	144	23	25	83	76	?	?
posterior free cervical	53	127	110	34	23	82	73	?	?
D1	53	118	107	38	28	104	120	110	22
A	46	128*	108*	?	25	?	?	?	?
B	?	?	?	?	20	114	107	100	?
C	?	?	?	?	?	212	205	110	?
D	39	110	89	22	16	208	213	100	?
E	88	122	55	11	9	198	184	57	14
F	48	110	86	19	17	183	196	86	?
G	?	?	?	?	?	?	?	?	?
H	59	110	77	?	?	?	?	?	?

Asterisk (*) denotes measurement as preserved (incomplete). See Fig 11 for dorsal vertebrae corresponding to positions A–H in first column below.

Nine free dorsal vertebrae are preserved in various states of completeness, and are identified by the presence of parapophyses on the neural arch, or otherwise by the presence of tall, narrow centra or conjoined zygapophyses [1]. Dorsal 1 is complete and the least distorted of all the vertebrae. The anterior and posterior faces of the centrum are dorsoventrally oblong, though absolutely and relatively smaller than their counterparts from the cervical series. There is no median ridge on the ventral surface of the centrum, as in *Chasmosaurus* [44]. A small (10 mm long x 5 mm tall) pleurocoel occurs on the dorsal of half of the lateral surface of the centrum. The parapophyses occur laterally at the base of the neural arch, approximately level with the neural canal, although the left parapophysis is more dorsally offset, which possibly resulted from plastic deformation. The neural canal is approximately teardrop shaped, as in some dorsal vertebrae of *Styracosaurus* [62] and *Triceratops* [63], but not *Chasmosaurus*, where it is oval [44]. The dorsomedially facing articular surfaces of the prezygapophyses are offset from one another by 104 degrees. Although the prezygapophyses remain distinct, there is no pronounced cleft or groove between them as there is in vertebra D1 of *Chasmosaurus* [44] and *Triceratops* ([63]: dorsal 3 –reinterpreted as dorsal 1 according to the identification key used here–of plate 5); rather the prezygapophyseal bases are conjoined as they are in *Centrosaurus* [64] and *Styracosaurus* [62]. A laterally opening pleurocoel, ~6 mm deep, occurs beneath each prezygapophysis. The midline keel for the attachment of the interspinous ligament [63] is strongly developed above the prezygapophyses. The dorsolaterally projecting transverse processes are shaped like an inverted ‘L’ in cross-section and expand distally to form rugose and laterally concave diapophyses. The distinct postzygapophyses face ventrolaterally. A median bony ridge descends beneath them to the dorsal margin of the neural canal. The tall neural spine is gently concave laterally and is slightly inclined posteriorly.

Only the relative positions of the remaining dorsals can be inferred with some degree of certainty (Table 3), based on morphological trends observed in other ceratopsids [1]. The centrum generally becomes dorsoventrally shorter and mediolaterally narrower posteriorly. The neural canal becomes more circular in outline and decreases in size. The pedicels of the neural arch become taller, and the zygapophyseal facets rotate to face either dorsally (prezygapophyses) or ventrally (postzygapophyses). The midline keel above the prezygapophyses reduces in size and eventually disappears. The neural spine becomes anteroposteriorly longer, although whether it eventually reduces in size again before the sacrum cannot be determined because of preservational incompleteness.

#### Ribs

At least 15 dorsal ribs are represented, based on the number of identifiable proximal rib ends. There are likely as many as 20 to 25 ribs actually present, although their completeness ranges from as much as 75% to as little as 10%. All ribs and rib fragments show signs of pre-depositional breakage, as there are green-stick bone fractures and isolated pieces that do not fit to any others recovered. These ribs represent various positions throughout the body, based on the variable shapes of the tuberculae, capitulae, and the angle at which the rib shafts extend from the rib heads. The shaft of an anterior dorsal rib extends ventrally at a nearly 90 degree angle from the rib head, while the shaft of a posterior dorsal rib has a much shallower angle, approaching 30 degrees.

Two anterior dorsal ribs are 70–75% complete. The rostral-most rib is likely the third dorsal rib. This rib shows a clear scapular facet, which is noted in other ceratopsids such as *Triceratops* and in sauropods [65, 66]. One other partial anterior dorsal rib shaft shows evidence of a scapular facet.

#### Humerus

**Fig 12:**
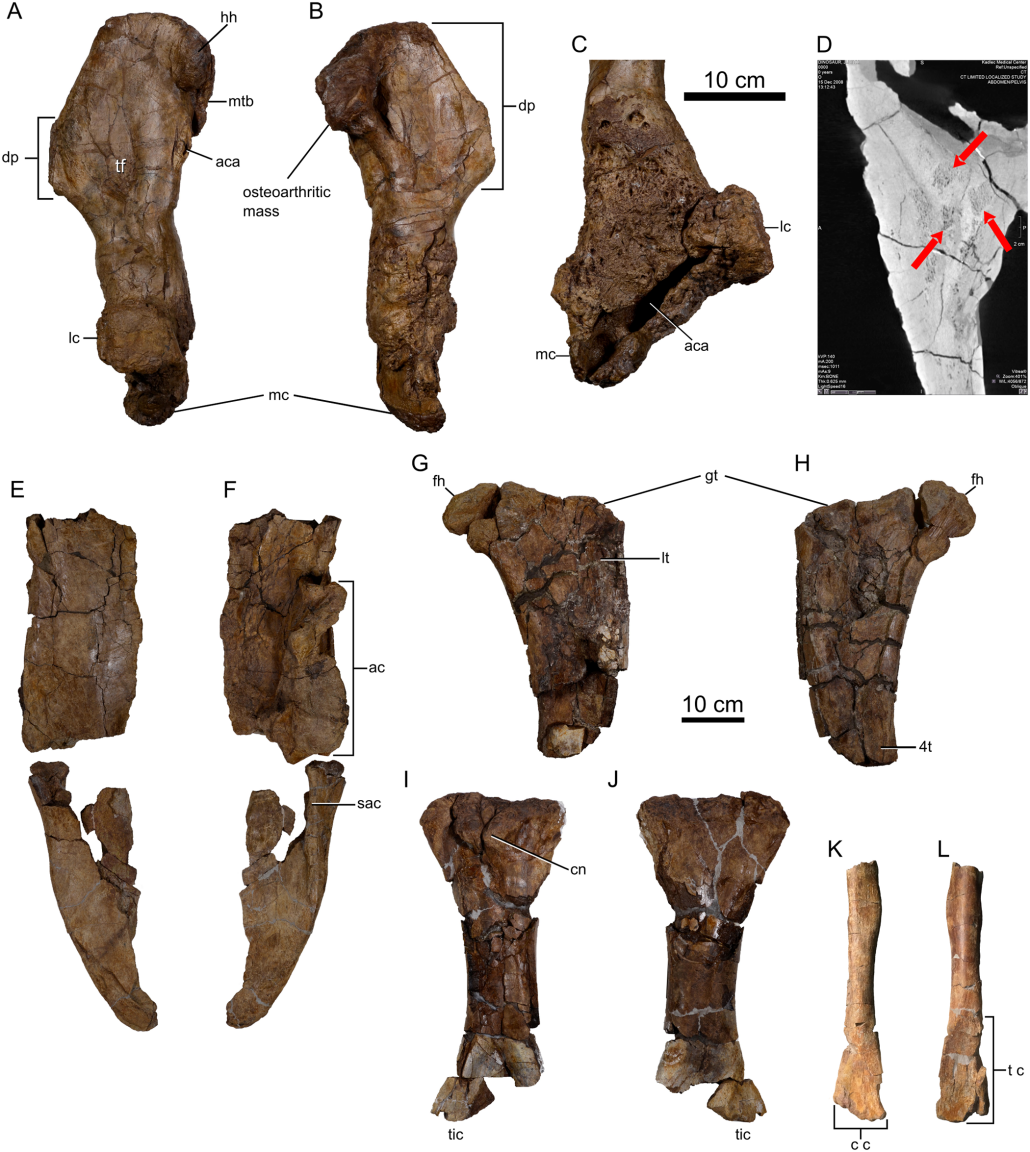
Appendicular elements of *Spiclypeus shipporum* gen et sp. nov. (CMN 57081). Left humerus in dorsal (A) and ventral (B) views; (C) detail of osteomyelitic infection at distal end of humerus; (D) CT image of distal left humerus showing bony sequestra (indicated by red arrows); left ilium in dorsal (E) and ventral (F) views; left femur in anterior (G) and posterior (H) views; left tibia in anterior (I) and posterior (J) views; left fibula in anterior (K) and posterior (L) views.

The left humerus (Fig 12A–12C) is the only element preserved from the forelimb. It is large and robust (Table 4), subequal in size to that of the largest specimen of *Chasmosaurus* (ROM 843). The hemispherical humeral head is rugose and directed posteromedially. The medial tubercle has buckled, obscuring the original outline; however, it was evidently well developed and likely triangular in outline, as in other ceratopsids [1]. A large (192 x 110 mm), anteromedially directed mass occurs opposite the humeral head and medial tubercle. The mass is roughened and irregular, consistent with a moderate degree of degenerative arthritis (EMI, pers. obs.). Marginal osteophytes occur medially and anteriorly, but no periarticular erosions are seen. The enlarged deltopectoral crest emerges anterolaterally from the humerus and extends mid-way down the length of the humeral shaft. The crest increasingly thickens in the direction of its distal apex. The crest is heavily striated posterolaterally where the deltoid musculature presumably would have attached. A deep groove circumscribes the apex of the deltopectoral crest, delimiting the area for the inferred insertion of the m. pectoralis, as in *Chasmosaurus* [44]. The shallow triceps fossa occurs posteriorly at the same level as the apex of the deltopectoral crest, nearer the humeral shaft. Opposite the deltopectoral crest, on the medial surface of the humerus, a deep and narrow fossa is bordered medially by a thin bony rim. The fossa opens proximally into a shallow, smooth-walled trough, and may represent localized osteomyelitis.

**Table 4:** Selected measurements from the appendicular skeleton of the holotype of *Spiclypeus shipporum* gen et sp. nov. (CMN 57081). All measurements are from the left side, reported in mm.

Measurement	Value
Humerus length from proximal end to distal end of lateral condyle	636
Humerus minimum circumference about shaft	349
Humerus maximum width across deltopectoral crest	206
Femur maximum preserved length	468
Femur minimum circumference about shaft	431
Tibia preserved length	538
Tibia minimum circumference about shaft	309
Fibula preserved length	366
Fibula minimum circumference about shaft	131

The severely pathological distal half of the humerus (Fig 12C) is highly inflated, and the distal condyles are rotated 90 degrees from their normal position. The bone surface is highly irregular and profusely covered by many pits (0.5–10 mm in diameter) and channels, particularly on the medial and anterior surfaces. Significant erosion of the distal end has resulted in the formation of a large (151 mm long x 24 mm wide x 42 mm deep) abscess cavity between the medial and lateral condyles, leaving little of the articular surface for the antebrachium intact. The destruction of the distal condyles is likely due to a combination of both degenerative osteoarthritis (similar to that occurring proximally) and chronic osteomyelitis. CT scans of region show disruption of the normal central marrow trabecular pattern with evidence of bony sequestra (fragments of trabecular bone floating in the central marrow space; Fig 12D), which are associated with chronic osteomyelitis.

#### Ilium

A partial left ilium, missing the preacetabular blade, is preserved (Fig 12E and 12F). The pubic and ischial penduncles have been crushed, obscuring their original morphology. Even so, they do not appear to differ greatly from those of other ceratopsids [1]. The pubic facet is anteroposteriorly elongate (93 mm long) and ~75% the length of the ovate ischial facet. The dorsal surface of the ilium is smooth and transversely wide, forming a distinctive shelf that laterally overhangs the acetabulum. The shelf deflects ventrally immediately posterior to the acetabulum to form a pronounced supraacetabular crest (“antitrochanter”), which is also seen in cf. *Anchiceratops* [67], *Triceratops* [46], and *Centrosaurus* [68]. The broken postacetabular process cannot be reattached to the remainder of the ilium; nevertheless, it appears to have projected no less than 450 mm behind the acetabulum. Viewed ventrally, the flat process gradually tapers posteriorly, as in *Triceratops* ([46]: fig 61) and *Centrosaurus* ([64]: fig 18), and does not form the blunt, parallel-sided termination seen in *Styracosaurus* ([62]: fig 21).

#### Femur

The proximal half of the left femur is preserved (Fig 12G and 12H). This element was the first found eroding from the edge of the discovery site, and it is in correspondingly poor condition. The femoral head projects dorsolaterally from the shaft, and the flat posterior face bears vertically trending striae, as in *Chasmosaurus* [44]. A saddle-shaped depression occurs on the dorsal surface of the femur, between the head and greater trochanter. The dorsal limit of the greater trochanter cannot be discerned because of poor preservation. Laterally, the greater trochanter is flat and weakly striated. The base of the lesser trochanter, which arises from the anterior face of the greater trochanter, can be determined, but the remainder of this feature is missing. The medially offset fourth trochanter is weakly developed, as in other ceratopsids [1], and projects from the posterior face of the femur. As preserved, it is 109 mm long, but the distal extremity is not preserved.

#### Tibia

The left tibia is partially preserved (Fig 12I and 12J). The entire dorsal surface is highly rugose, suggesting that it was capped by thick cartilage in life. The transversely narrow cnemial crest extends above the dorsal limit of the shaft, and appears as a scalene triangle in lateral view, which contrasts with the more rounded crest of other ceratopsids ([1]: fig 23.7). The fibular condyle occurs posterolateral to the cnemial crest. It projects laterally for a short distance, and then curls anteriorly. The rugose lateral surface of the condyle is continuous with the dorsal rugosity of the fibula. The proximal end of the fibula expands posteriorly so that it is anteroposteriorly longer than transversely wide. Although incomplete, the distal end of the fibula is likewise anteroposteriorly longer than transversely wide, with a pendant articular surface for the calcaneum anteriorly. Maidment and Barrett [44] noted a similar morphology in *Chasmosaurus*, and argued that the tibia must have been distorted because the distal end is normally transversely wider than anteroposteriorly long in ornithischians.

#### Fibula

A single left fibula is partially preserved (Fig 12K and 12L). Where the proximal end has broken away, the shaft is subtriangular in cross section. Distally, the fibula flares slightly transversely where it overlaps the tibia. The distal articular facet for the tibia is approximately triangular in outline and faintly striated.

### Osteohistological analysis

**Fig 13:**
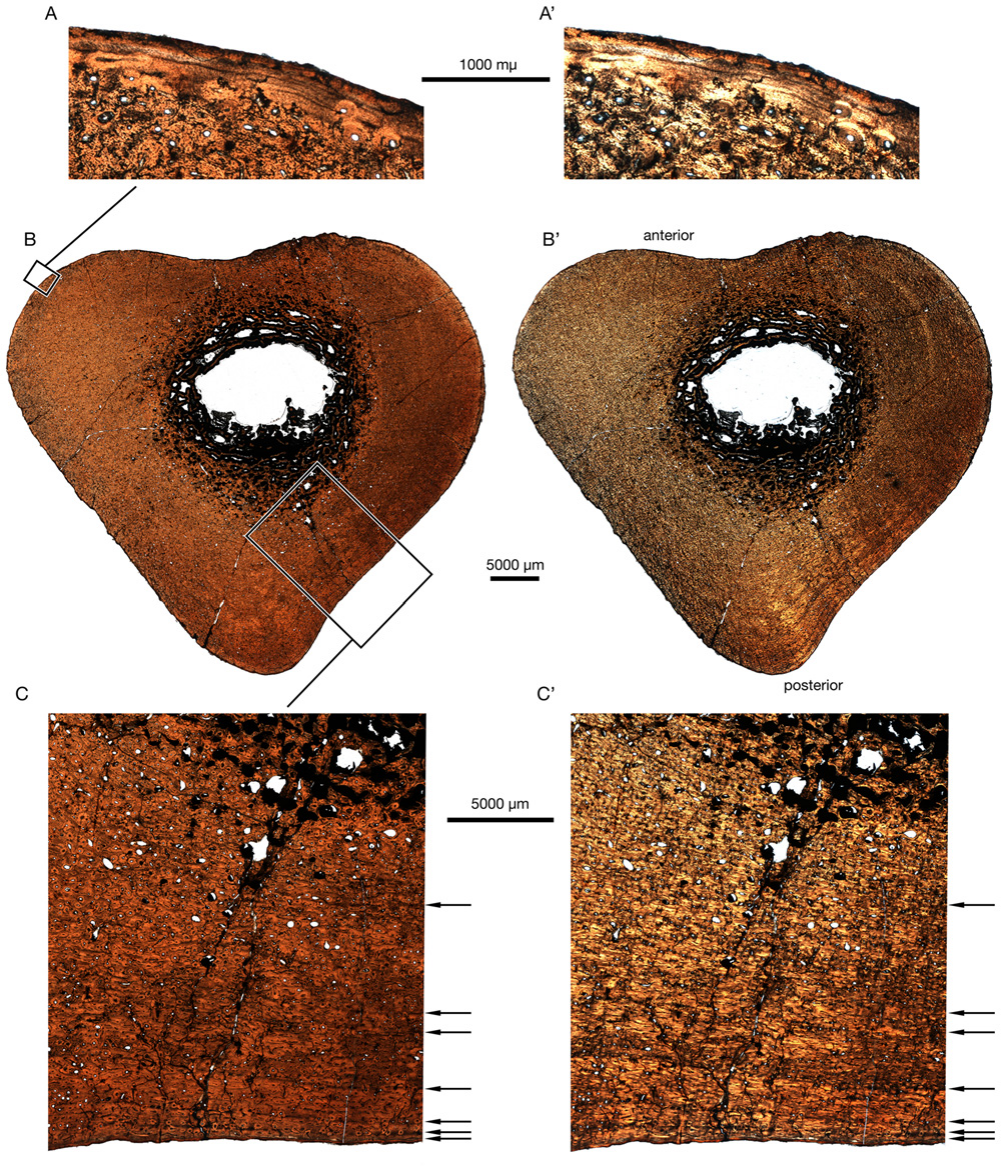
Osteohistology of the fibula in *Spiclypeus shipporum* gen et sp. nov. (CMN 57081) in transverse section under normal (left) and polarized (right, prime designations) light. (A) Close-up of inset in (B) showing the periosteal surface on the anterior side of the bone and the development of poorly vascularized, highly organized primary bone tissue of the external fundamental system; (B) full transverse section of fibula; and (C) close-up of inset in (B) exhibiting the least amount of remodelling and showing growth marks (indicated by arrows) in the primary bone tissue.

The fibula was transversely sectioned above the midshaft, approximately one-third of the total length from the proximal end (Fig 13). The cortex varies in thickness around the small, sub-circular medullary cavity which itself is surrounded by a small amount of cancellous bone (Fig 13B). The majority of the cortex consists of densely packed and overlapping secondary osteons formed by extensive secondarily remodelling. Remodelling is particularly well developed in the lateral region of the bone, where it completely obliterates the primary bone tissue. There is less remodelling posteriorly and medially, where the fibula is adjacent to the tibia. Here, some of the primary bone is visible, but it is nonetheless disrupted by secondary osteon development, making the identification of the primary tissue and growth marks challenging, especially in the deep cortex (Fig 13B). The primary cortical tissue consists largely of parallel-fibered matrix in the mid-cortex and outwards toward the periosteum. Primary vascularization is dominated by longitudinal canals, with relatively frequent but short, typically circumferential, anastomoses. Vascularity appears to decrease moderately from the mid to outer cortex. Seven growth marks (Lines of Arrested Growth) can be traced through much of the medial side of the section, but zones or annuli in vascular patterns are difficult to identify in the region of primary bone that is visible. The spacing of growth marks generally decreases towards the periosteum, with a series of three closely spaced LAGs present near the external surface of the bone (Fig 13C). The outermost cortex, where not obscured by secondary remodelling, is comprised of poorly vascularized, highly organized bone and appears to form an external fundamental system (Fig 13A). This signals the cessation of significant peripheral bone growth and suggests that CMN 57081 had reached somatic maturity. Determination of the number of growth marks within the EFS is not possible, although it seems likely that there are at least two in some regions, but these cannot be traced consistently. Retrocalculation of the earliest growth marks is difficult given the shape of the bone and the extent of the medullary cavity. However, we estimate that CMN 57081 was likely at least 10 years old at death, and had reached its growth asymptote.

### Phylogenetic analysis

**Fig 14:**
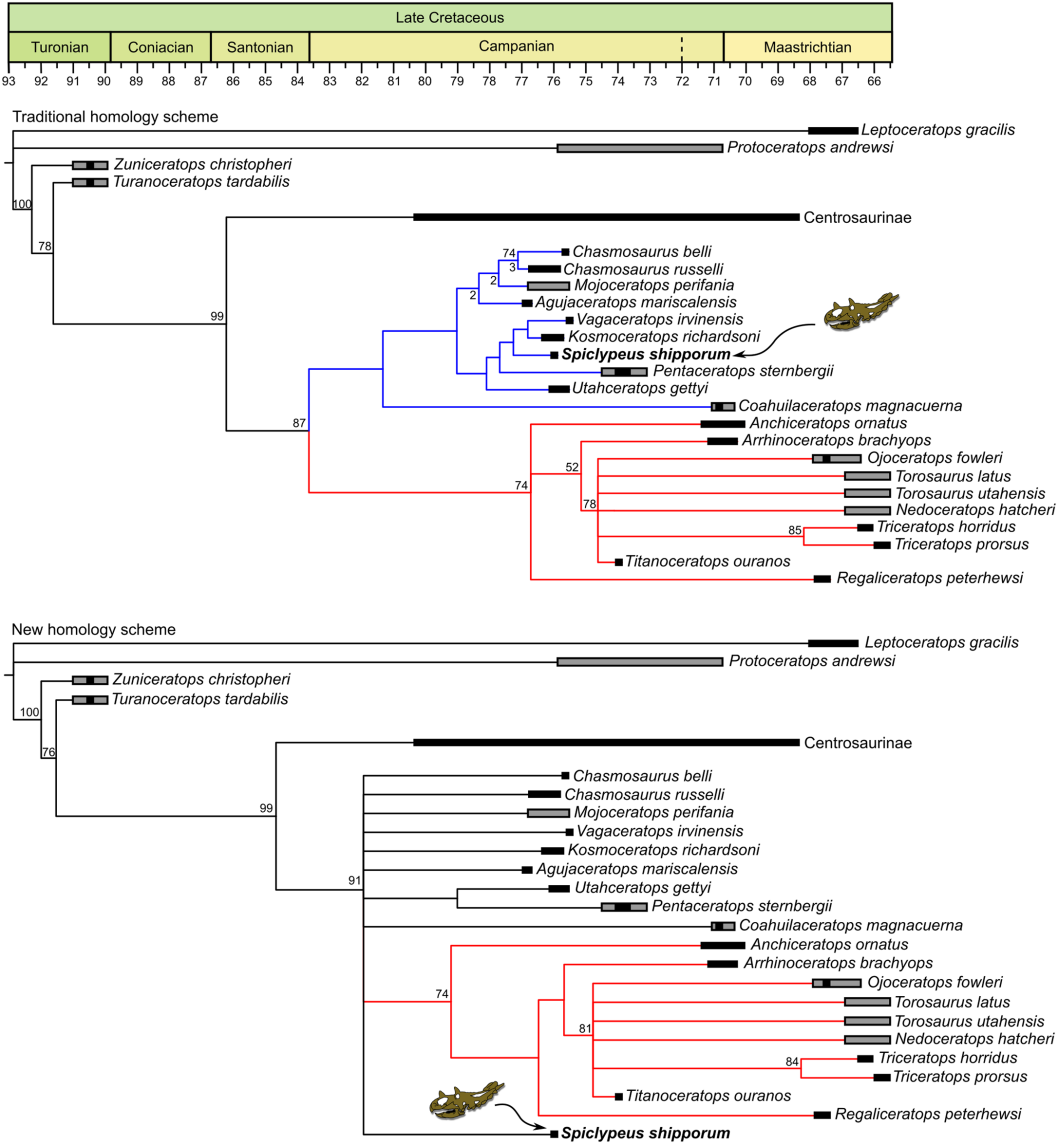
Cladograms showing evolutionary relationships within Chasmosaurinae. Top: reduced strict consensus tree assuming traditional homology scheme for epiparietals (tree length = 332 steps, consistency index = 0.5873, retention index = 0.7345); Bottom: reduced strict consensus tree assuming new homology scheme for epiparietals (tree length = 439 steps, consistency index = 0.4487, retention index = 0.5346). Blue branches indicate ‘*Chasmosaurus* clade’ described in text; red branches indicate ‘*Triceratops* clade’. Numbers above nodes indicate bootstrap values >50%; numbers beneath nodes indicate Bremer support values >1.

The cladistic parsimony analysis using the traditional epiparietal homology scheme returned 28 most parsimonious trees (MPTs) of 320 steps each. The analysis using the new epiparietal homology scheme returned 91 MPTs of 321 steps each. The resulting strict consensus trees were not particularly well-resolved, especially in the latter case (S1 Fig). For this reason, we pruned the wildcard taxa *Bravoceratops* and *Eotriceratops*, previously identified by Mallon et al. [36], from the MPTs *a posteriori* and calculated reduced strict consensus trees, resulting in slightly improved resolution for the analysis using the new homology scheme (Fig 14).

The analysis using the traditional homology scheme (Fig 14, top) is better resolved than that of Brown and Henderson ([34]: fig S1). Chasmosaurinae exhibits a deep split, resulting in a *Chasmosaurus* clade (*Chasmosaurus* > *Triceratops*) and a *Triceratops* clade (*Triceratops* > *Chasmosaurus*), in accordance with recent findings [25, 34, 36, 69]. Bootstrap support for each of these clades is weak (49%) to moderate (74%), respectively. The *Chasmosaurus* and *Triceratops* clades are each supported by three unambiguous synapomorphies primarily relating to the snout and parietosquamosal frill (S1 Appendix). *Spiclypeus* falls within the *Chasmosaurus* clade, as the sister taxon to the group *Kosmoceratops* + *Vagaceratops*. Bootstrap support for the *Spiclypeus* (*Kosmoceratops* + *Vagaceratops*) clade is low (32%). The nine synapomorphies that unite this clade primarily relate to the snout, postorbital horncores, and the parietosquamosal frill and its epiossifications (S1 Appendix).

The reduced strict consensus tree using the new homology scheme (Fig 14, bottom) is more poorly resolved than that assuming the traditional homology scheme (and that of Brown and Henderson [34]) in that the constituent taxa of the *Chasmosaurus* clade are reduced to a polytomy at the base of Chasmosaurinae. The *Triceratops* clade is recovered with the same topology as that using the traditional homology scheme, and maintains good bootstrap support (74%). Fourteen unambiguous synapomorphies unite *Anchiceratops* with the remainder of the *Triceratops* clade, primarily relating to the snout and parietosquamosal frill (S1 Appendix).

## Discussion

### Taxonomic validity of *Spiclypeus shipporum*

The first recognized ceratopsid was ‘*Ceratops montanus*’ (USNM 2411) from the upper JRF, known only from a pair of postorbital horncores (Fig 6G) and partial occiput [3]. The holotype locality was described as the “northwestern slope near the summit, about 300 yards from the point of the first hogback that projects into the valley of Cow Creek from the west, just below where the old Cow Island and Fort Benton freight road descends into the valley of Cow Creek, about 10 miles above the confluence of that stream with the Missouri River” [46]. This places the site ~53 km away from that of CMN 57081, and both sites are among the few located along the Missouri River to yield ceratopsid material. Marsh [3] initially illustrated the horncores of ‘*Ceratops*’ as projecting anterodorsally, but Hatcher et al. [46] subsequently showed that they instead projected dorsolaterally. In fact, the horncores closely resemble those of CMN 57081, which are just 20 mm longer (Fig 6A–6F). Given the similarity between the two specimens, and their geographic and stratigraphic proximity, it is possible that they represent the same taxon. If so, this finding would be significant because ‘*C*. *montanus*’ has long been considered a nomen dubium [1, 5, 7, 16], owing to its scant remains, and the assignment of new fossil material would help clarify one of the longest standing mysteries in horned dinosaur palaeontology. The issue hinges on whether the postorbital horncores of ‘*C*. *montanus*’ are diagnostic to the species level, which is typically not the case among ceratopsids [5], and where the size and orientation of the horncores is known to vary with age in other chasmosaurines [70, 71].

Two other ceratopsid species, the centrosaurine *Albertaceratops* [5] and the chasmosaurine *Kosmoceratops* [35], have also been described as having elongate, dorsolaterally projecting postorbital horncores. Those of the *Kosmoceratops* holotype are especially similar to those of ‘*Ceratops*’ in size and shape (Table 1; Fig 6G). The preservation of a growth series of *Albertaceratops* reveals that the postorbital horncores grew longer with age, originating as the short pyramidal elements (e.g., WDCB-MC-001) typically seen in the adults of more derived centrosaurines, and eventually reaching lengths of at least 342 mm (e.g., TMP 2002.003.0036; Fig 6I). The horncores of the ‘*Ceratops*’ holotype fall somewhere between these two extremes, and might conceivably represent a growth stage of *Albertaceratops*, which occurs slightly lower in section within the same formation (Fig 2). Therefore, we concur with previous studies [1, 5, 7, 16] that the postorbital horncores of ‘*Ceratops*’ are non-diagnostic, and that the monospecific genus is a nomen dubium. It is possible—even likely, given their close stratigraphic and geological association—that ‘*Ceratops*’ and CMN 57081 are the same species, but without conclusive evidence for such, it is preferable to erect a new species for CMN 57081.

### Taxonomic validity of *Pentaceratops aquilonius*

**Fig 15:**
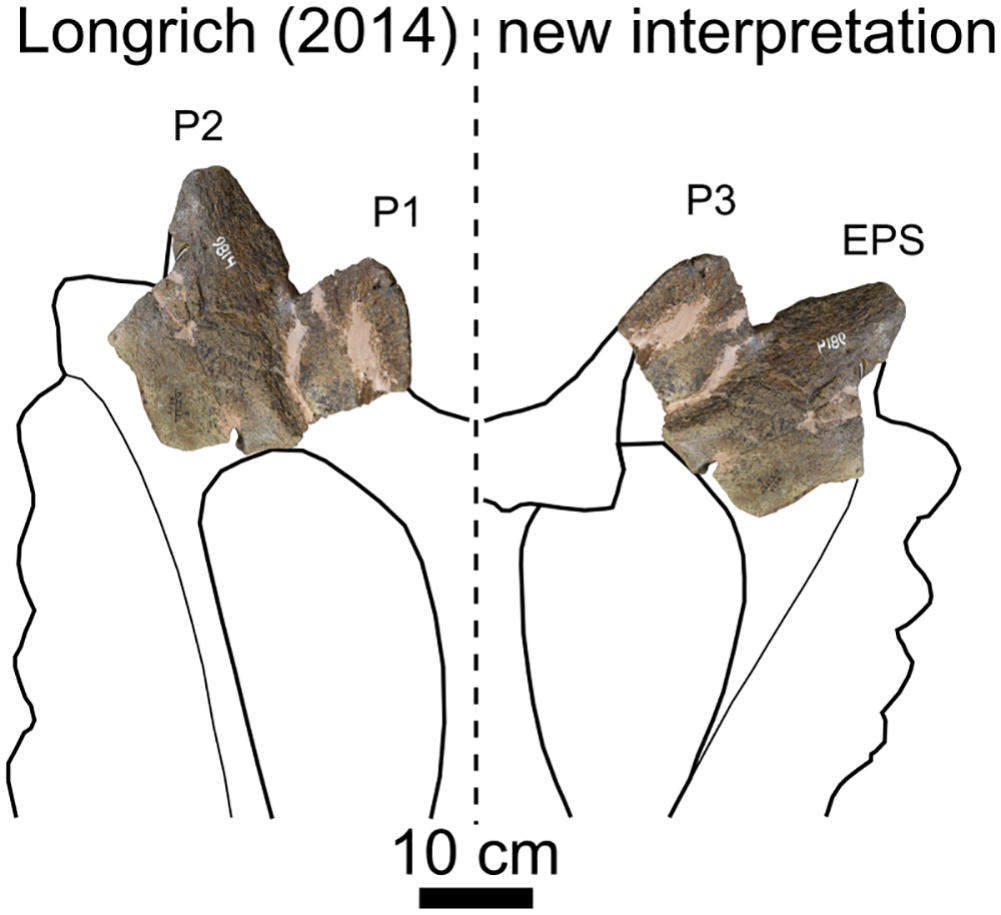
Interpretation of CMN 9813 (paratype of ‘*Pentaceratops aquilonius’*) in Longrich [72] (left) and the present study (right). In the right image, the specimen is mirrored about the midline to facilitate comparison. Note the differences in assumed epiossification homology. [Planned for column width].

Longrich [72] established the new chasmosaurine species *Pentaceratops aquilonius* based on a partial parietosquamosal frill (CMN 9813), collected in 1937 by C. M. Sternberg from the Lethbridge Coal Zone near Manyberries, Alberta, and initially attributed to *Anchiceratops* [73, 74]. Although the holotype frill closely resembles the upper Campanian/lower Maastrichtian *Anchiceratops* (particularly the holotype of *A*. *‘longirostris*’; see Mallon et al. [57]) in the size and shape of its preserved epiossifications, Longrich noted that the specimen is unlikely to pertain to this genus because of its much earlier occurrence (~4 Myr), and because the parietal fenestrae more closely approach the parietal-squamosal suture as in *Pentaceratops*. Longrich assigned an additional parietal fragment (CMN 9814, the paratype) to *P*. *aquilonius*, also collected in 1937 nearly 16 km away from the holotype locality, at approximately the same stratigraphic horizon. According to Longrich’s interpretation, the fragment bears a dorsally directed P1 epiparietal, and a posteriorly directed P2 epiparietal lying entirely within the plane of the frill (Fig 15, left). Assuming the paratype to be attributable to same species as the holotype, Longrich [72] diagnosed *P*. *aquilonius* as follows: weak median emargination of parietal, with lateral rami meeting at an angle of ~90 degrees, and U-shaped emargination not strongly extended anteriorly; P1 projects dorsally, rather than anteriorly; lateral epiossifications <100 mm in length; posterior bar of parietal anteroposteriorly broad relative to *P*. *sternbergii* or *Utahceratops gettyi*.

The discovery of *Spiclypeus shipporum* casts doubt on the validity of *P*. *aquilonius*. The *P*. *aquilonius* paratype quite closely matches *S*. *shipporum* in the morphology of the posterior parietal. What Longrich [72] labelled as epiparietals P1 and P2 closely resemble epiparietal P3 and the EPS of *S*. *shipporum* (Fig 15, right). Notably, what we interpret as the incomplete P3 epiparietal is slightly twisted and medially thickened where it presumably once would have merged with the coossified P2 epiparietal. Given this interpretation of the paratype, the free posterior parietal border identified by Longrich (which he presumed was once capped by the P3 epiparietal) instead becomes the squamosal contact, an interpretation that is consistent with the lack of scalloping or scarring expected of an epiossification attachment site.

Given this reinterpretation of the *P*. *aquilonius* paratype, the relationship of this specimen to the holotype is obscured because the morphology of epiparietal P3 (Longrich’s P2) no longer corresponds between both specimens. Whereas the P3 epiparietal of the paratype is dorsally directed, it is posteriorly directed in the holotype ([72]: fig 3). This inconsistency is conceivably attributable to species-level differences, in which case, the holotype of *P*. *aquilonius* is distinct and the species remains valid. However, the small size of the specimen (corresponding to a frill just ~100 cm long; see Longrich [72]) bespeaks a relatively immature individual, and it is plausible that the development of the frill epiossifications would have changed with age to become more like that of the paratype, which comes from a larger individual. Such changes in frill ornamentation, including the growth, reorientation, and fusion of the epiossifications, are common among ceratopsids [71, 75]. These doubts are difficult to assuage, given the poor quality of the material referred to *P*. *aquilonius*, and for this reason we consider the species a nomen dubium.

### Pathologies of CMN 57081

It is evident from the various pathologies that riddle the frill and humerus of CMN 57081 that the animal was in poor health before it died. Tanke and Farke [47] made the case that squamosal fenestrae in chasmosaurines usually result from bone resorption under normal conditions; however, the association of squamosal fenestrae in CMN 57081 with abscess cavities and drainage tracts suggests that the fenestrae may be pathological in origin. The etiology of the pathology is unknown, but the largest squamosal fenestra conceivably resulted from intraspecific combat, as its general shape and location on the animal is consistent with a blow from the laterally pointing postorbital horncores of a conspecific [76, 77]. The wound would later have become infected, leading to remodelling of the bone.

The distal half of the humerus exhibits rough bony remodeling and the presence of an abscess cavity between the condyles, consistent with a chronic infection. One can only speculate as to the infectious organism responsible; however, chronic tuberculous infections have a similar appearance in modern humans. Bacterial infections would typically rapidly weaken the bone, leading to bone destruction and pathologic fractures. This is not seen in CMN 57081, suggesting that the animal was walking around for a prolonged period of time, giving the bone time to respond to the infection via hypertrophic remodeling. This would suggest infection by a more indolent organism such as tuberculosis or perhaps a fungus. Despite these impairments, skeletochronology indicates that the animal lived at least 7–10 years, attesting to its robust constitution.

### Dinosaur assemblage of Judith River Formation

Despite its long collecting history (e.g., [14, 17, 18, 78–80]), the dinosaur assemblage of the JRF remains relatively poorly understood, presently known from just nine reasonably diagnostic species, and several other microfossil taxa (Fig 2). By comparison, the time-equivalent Belly River Group of Alberta has yielded ~55 distinct species [9], and the Kaiparowits Formation of Utah has yielded ~16 [81].

However insufficiently sampled the dinosaur assemblage of the JRF, the discovery of *Spiclypeus* highlights both the taxonomically unique and temporally dynamic nature of the assemblage. Correlation and dating of the strata of the JRF throughout Montana [10, 13, 23] provide a useful framework for investigating faunal turnover within the formation (Fig 2), although dating accuracy remains problematic (see [10] and [21] for details). The oldest of the JRF strata are best exposed near Rudyard (Kennedy Coulee) in north central Montana. These strata date to between 79.5 ± 0.2 Ma and 79.1 ± 0.2 Ma, based on ^40^Ar/^39^Ar analysis of biotite and sanidine crystals from two local bentonites ([23]; recalibrated in [10]), and fossil occurrences are generally attributable to the lower McClelland Ferry Member (sensu Rogers et al. [10]), correlative to the uppermost Foremost Formation (Taber Coal Zone and Herronton Sandstone) and lowermost Oldman Formation in Alberta [21]. The ceratopsids *Medusaceratops* [7] and *Albertaceratops* [5], the hadrosaurid *Probrachylophosaurus* [21], and the pachycephalosaurid *Colepiocephale* [22] occur within the upper muddy unit of the Kennedy Coulee area, which corresponds to Unit 1 of the Oldman Formation (~79.1 Ma). The hadrosaurid *Brachylophosaurus goodwini* [82] also originates from these strata, but is most recently considered to be an indeterminate brachylophosaurin [21]. The ceratopsid *Judiceratops* [6] likewise occurs in Kennedy Coulee, but its stratigraphic position is not precisely known. The holotypes of *Wendiceratops* and *Albertaceratops* were collected in lithostratigraphically and chronostratigraphically equivalent sediments of the lowermost Oldman Formation just across the international border in Alberta [83].

Exposures of the JRF near Malta, Montana, ~200 km ESE of Kennedy Coulee, have yielded abundant remains attributable to *Brachylophosaurus canadensis* [21, 84, 85]. Exposures in this area are more distal along the depositional wedge and closer to the Western Interior Seaway. The tan coloured, quartz-rich sandstones exposed here are attributable to the middle McClelland Ferry Member, equivalent to the Comrey Sandstone Zone (Unit 2) of the middle Oldman Formation in Alberta, and date to at least ~77.76 Ma [21].

Approximately 250 km SW of Malta, the JRF is well exposed near the Musselshell River of Wheatland and Golden Valley counties. Fossil locales here typically occur in sandy fluvial deposits within ~70 m of the Parkman Sandstone Member [20], and likely also pertain to the lower McClelland Ferry Member (R. Rogers, pers. comm., 2016). Dinosaurs reported from these deposits are known primarily from microfossil bonebeds, and include the dromaeosaurids *Dromaeosaurus albertensis* and *Saurornitholestes langstoni*, the troodontid *Troodon formosus*, *Richardoestesia gilmorei* and another indeterminate theropod [19], and tyrannosaurids, hadrosaurids, pachycephalosaurids, nodosaurids, and ceratopsids [20]. The holotype of the ceratopsid *Avaceratops lammersi*, which is known from a reasonably complete skeleton, originates from the Careless Creek Quarry, which occurs locally [4, 20]. The age of the beds in this region is poorly constrained, but the strata likely date to no younger than ~76.24 Ma, which is the age of the upper boundary of the McClelland Ferry Member in the JRF type area [10].

The type area for the JRF occurs within the Upper Missouri River Breaks National Monument [12], where the formation is exposed in its entirety. This is also the region where the first fossil vertebrates were collected from the JRF in the latter half of the 19^th^ century. The earliest expeditions occurred near the confluence of the Judith and Missouri rivers, where the McClelland Ferry and lowermost Coal Ridge members are well exposed ([10]; R. Rogers, pers. comm., 2016). Here, F. V. Hayden found remains of the tyrannosaurid *‘Deinodon horridus’*, the troodontid *Troodon formosus*, the nodosaurid *‘Palaeoscincus costatus’*, and the hadrosaurid *‘Trachodon mirabilis’* [17, 78]. Subsequent work in the area by E. D. Cope and assistant C. H. Sternberg uncovered new species, including the tyrannosaurids *‘Aublysodon lateralis’*, *‘Laelaps incrassatus’*, *‘L*. *falculus’*, and *‘L*. *hazenanius’*, the dromaeosaurids *‘L*. *laevifrons’* and *Zapsalis abradens*, the troodontids *‘L*. *explanatus’*, *‘L*. *cristatus’*, and *Paronychodon lacustris*, the ceratopsids *‘Dysganus encaustus’*, *‘Dy*. *haydenianus’*, *‘Dy*. *bicarinatus’*, and *‘Dy*. *peiganus’*, and the hadrosaurids *‘Diclonius pentagonus’*, *‘Di*. *perangulatus’*, and *‘Di*. *calamarius’* [14, 18]. Most of these species are now considered *‘nomina dubia’* [1, 86–88].

Further downstream on the Missouri River, near the area of Cow Island, Cope and Sternberg later found partial cranial remains of the indeterminate centrosaurines *‘Monoclonius crassus’*, *‘M*. *sphenocerus’*, *‘M*. *recurvicornis’*, and *‘M*. *fissus’* [15]. The uppermost McClelland Ferry and lower Coal Ridge members are exposed here, and it seems likely that these problematic taxa are restricted to the latter member, given its more extensive exposure ([10]; R. Rogers, pers. comm., 2016). Microfossil bonebeds are especially common elsewhere in the lower third of the Coal Ridge Member, and contain evidence of tyrannosaurids, troodontids, ankylosaurs, ceratopsids, and hadrosaurids [13]. The ceratopsids *Spiclypeus* and *Mercuriceratops* [8] are likewise known from this interval. The microfossil material described by Sahni [12] is from the upper third of the Coal Ridge Member, and includes tyrannosaurids, troodontids, ankylosaurids, pachycephalosaurids, ceratopsids, “hypsilophodontids”, and hadrosaurids [13]. These date to no later than ~75.21 Ma, which is the age of the upper boundary of the Coal Ridge Member [10]. Exposures of the Coal Ridge Member near Havre contain microsite bonebeds and hadrosaurid eggs and embryos [89].

To date, five of the nine well-described species from the JRF are known exclusively from the formation, with the remainder known also from time-equivalent strata in Alberta, Canada [80, 90]. Material from Alberta previously assigned to ‘*Pentaceratops aquilonius*’ may strengthen this biogeographic link, should it prove to be closely allied to *Spiclypeus*. With the exception of some dubious tooth taxa, there are, as yet, no species from Montana that also occur in time-equivalent strata further south (e.g., Kaiparowits Formation, Utah; Fruitland Formation, New Mexico; Aguja Formation, Texas), according with previous suggestions that there existed a dispersal barrier somewhere between Montana and Utah [35, 90, 91]. However, the close affinity between *Spiclypeus* from Montana and *Kosmoceratops* from Utah suggests that some ephemeral geographic connection was present. Longrich [72] argued that *Kosmoceratops* ranged as far north as Alberta, but this was recently disproved by Campbell et al. [37].

### Evolution of Chasmosaurinae

The present study is part of a growing body of work [25, 34, 36, 69] finding cladistic support for a deep split within Chasmosaurinae, yielding *Chasmosaurus* and *Triceratops* as the terminal end-members of their respective subclades (although the present analysis using the new homology scheme is ambiguous in this respect). Brown and Henderson [34] correctly pointed out that such a topology implies a long ghost lineage for the ‘*Triceratops* clade’, extending from the late Maastrichtian into the late Campanian, a duration of ~6 Myr. However, we note that this interpretation is contingent on the classification of the late Campanian to early Maastrichtian *Titanoceratops*, *Anchiceratops*, and *Arrhinoceratops* as members of the ‘*Triceratops* clade’, which is debated [25, 36, 69, 70]. Was this not the case, the ‘*Triceratops* clade’ would become synonymous with Triceratopsini (sensu Longrich [92]), and entirely relegated to the mid- to late Maastrichtian, greatly reducing the length of the implied ghost lineage and underscoring the role that adaptive radiation may have played in the evolution of the clade. Regardless, we note that there is presently considerable debate concerning the relationships within Chasmosaurinae, with some [35, 72] arguing for a generally pectinate topology in which *Chasmosaurus* is basal and *Triceratops* is most derived. Statistical support for this or any other topology is currently weak, likely reflecting issues of missing data and character conflict. Besides determined fieldwork and the pursuit of more complete specimens, we would likewise encourage the search for new and phylogenetically informative characters, particularly in the braincase (e.g., [25]) and postcranium (e.g., [39, 44]), which are traditionally overlooked. Explicit and illustrated character state definitions would also help to ensure compatibility between studies, which is presently concerning.

This study recovers *Kosmoceratops* and *Vagaceratops* as sister taxa, united by an anteroposteriorly abbreviated frill, small and caudally placed parietal fenestrae, and the presence of 10 anteriorly curled epiossifications on the posterior margin of the frill. This relationship is in keeping with most recent cladistic findings [25, 34–36, 69], although some [37, 59, 72] maintain that *Vagaceratops* is more closely allied to *Chasmosaurus*. Regardless, the anteriorly curled epiossifications of *Kosmoceratops* and *Vagaceratops* are highly distinctive among chasmosaurines, and no explanation has yet been provided as to how such an elaborate condition was achieved. The recovery of *Spiclypeus* as the sister taxon to *Kosmoceratops* + *Vagaceratops* suggests that the anterior curling of the posterior frill epiossifications occurred in a step-wise fashion, at least on the line leading to *Kosmoceratops*, with the medialmost epiparietals curling anteriorly first, followed by those epiossifications further laterally. The onset of this process evidently preceded the anteroposterior abbreviation of the frill and constriction of the parietal fenestrae, as these characters occur in their primitive state in *Spiclypeus*. Despite the transitional nature of *Spiclypeus* in these respects, the taxon was approximately equivalent in time to *Kosmoceratops* ([93]: fig 14]), and so could not have been ancestral to it.

## Supporting Information

S1 Appendix Unambiguous synapomorphies uniting the ‘*Chasmosaurus*’ (blue), ‘*Triceratops*’ (red), and *Spiclypeus* (*Vagaceratops* + *Kosmoceratops*) clades shown in Fig 14 under different homology schemes. (DOCX)

S1 Fig Cladograms showing evolutionary relationships within Chasmosaurinae. Left: strict consensus tree assuming traditional homology scheme for epiparietals (tree length = 332 steps, consistency index = 0.5873, retention index = 0.7345); right: strict consensus tree assuming new homology scheme for epiparietals (tree length = 439 steps, consistency index = 0.4487, retention index = 0.5346). Blue branches indicate ‘*Chasmosaurus* clade’ described in text; red branches indicate ‘*Triceratops* clade’. Numbers above nodes indicate bootstrap values >50%; numbers beneath nodes indicate Bremer support values >1.
(TIF)

S1 File Three-dimensional model for the skull reconstruction of Spiclypeus shipporum gen. et sp. nov. (CMN 57081). (PDF)

S2 File Nexus (.nex) file used for analysis assuming traditional epiparietal homology scheme. (NEX)

S3 File Nexus (.nex) file used for analysis assuming new epiparietal homology scheme. (NEX)

## Abbreviations

CMN: Canadian Museum of Nature, Ottawa, Ontario
ROM: Royal Ontario Museum, Toronto, Ontario
TMP: Royal Tyrrell Museum of Palaeontology, Drumheller, Alberta
UMNH: Utah Museum of Natural History, Salt Lake City, Utah
USNM: United States National Museum, Washington, D.C.
WDCB: Wyoming Dinosaur Center, Thermopolis, Wyoming
ac: acetabulum
aca: abscess canal
af: adductor fossa
an c: angular contact
aofe: antorbital fenestra
ar c: articular contact
c c: calcaneum contact
cn: cnemial crest
corp: coronoid process
d c: dentary contact
dp: deltopectoral crest
ej: epijugal
ej c: epijugal contact
EPS: epiparietosquamosal
fh: femoral head
gt: greater trochanter
hh: humeral head
itf: infratemporal fenestra
ja: jaw articulation
j c: jugal contact
lc: lateral condyle
lt: lesser trochanter
mc: medial condyle
mec: Meckelian canal
mimx: medial inflection of maxilla
mtb: median tubercle
mx c: maxilla contact
n c: nasal contact
ns: narial strut
o: orbit
on: otic notch
P1–P3: epiparietal loci P1–P3
pf: parietal fenestra
pfo: palatal fossa
pmx: premaxilla
pmxf: premaxillary fossa
pr: primary ridge
pra c: prearticular contact
ptf: pterygoid flange
q c: quadate contact
qj: quadratojugal
qj c: quadratojugal contact
r: rostral
r c: rostral contact
s: sinus
S1–S7: episquamosal loci S1–S7
sac: supraacetabular crest
sa c: surangular contact
sf: septal flange
spl c: splenial contact
sq: squamosal
sq c: squmosal contact
sqf: squamosal fenestra
t c: tibia contact
tf: triceps fossa
tic: internal condyle of tibia
tp: triangular process
tr: tooth row
trr: transverse ridge
wf: wear facet
4t: fourth trochanter

## References

[1] Dodson P, Forster CA, Sampson SD. Ceratopsidae In: Weishampel DB, Dodson P, Osmólska H, editors. The Dinosauria. 2^nd^ ed Berkeley: University of California Press; 2004 pp. 494–513.

[2] Farlow JO, Dodson P. The behavioral significance of frill and horn morphology in ceratopsian dinosaurs. Evolution. 1975;2: 353–361. 10.1111/j.1558-5646.1975.tb00214.x 28555861

[3] Marsh OC. A new family of horned Dinosauria, from the Cretaceous. Am J Sci, series 3. 1888;36: 477–478.

[4] Dodson P. *Avaceratops lammersi*: a new ceratopsid from the Judith River Formation of Montana. Proc Acad Nat Sci Phila. 1986;138: 305–17.

[5] Ryan MJ. A new basal centrosaurine ceratopsid from the Oldman Formation, southeastern Alberta. J Paleontol. 2007;81: 376–396.

[6] Longrich NR. *Judiceratops tigris*, a new horned dinosaur from the middle Campanian Judith River Formation of Montana. Bull Peabody Mus Nat Hist. 2013;54: 51–65.

[7] Ryan MJ, Russell AP, Hartman SC. A new chasmosaurine ceratopsid from the Judith River Formation, Montana In: Ryan MJ, Chinnery-Allgeier BJ, Eberth DA, editors. New perspectives on horned dinosaurs: the Royal Tyrrell Museum Ceratopsian Symposium. Bloomington: Indiana University Press; 2010 pp. 181–188.

[8] Ryan MJ, Evans DC, Currie PJ, Loewen MA. A new chasmosaurine from northern Laramidia expands frill disparity in ceratopsid dinosaurs. Naturwissenschaften. 2014;101: 505–512. 10.1007/s00114-014-1183-1 24859020

[9] Brown CM, Ryan MJ, Evans DC. A census of Canadian dinosaurs: more than a century of discovery In: Bininda-Emonds ORP, Powell GL, Jamniczky HA, Bauer AM, Theodor J, editors. All Animals are Interesting: A Festschrift in Honour of Anthony P. Russell. Oldenburg, Germany: BIS Verlag; 2015 pp. 151–209.

[10] Rogers RR, Kidwell SM, Deino AL, Mitchell JP, Nelson K, Thole JT. Age, correlation, and lithostratigraphic revision of the Upper Cretaceous (Campanian) Judith River Formation in its type area (north-central Montana), with a comparison of low- and high-accommodation alluvial records. J Geol. 2016;124: 99–135.

[11] Rogers RR. Sequence analysis of the Upper Cretaceous Two Medicine and Judith River formations, Montana: nonmarine response to the Claggett and Bearpaw marine cycles. J Sediment Res. 1998;68: 615–631.

[12] Sahni A. The vertebrate fauna of the Judith River Formation, Montana. Bull Am Mus Nat Hist. 1972;147: 321–412.

[13] Rogers RR, Brady ME. Origins of microfossil bonebeds: insights from the Upper Cretaceous Judith River Formation of north-central Montana. Paleobiology. 2010;36: 80–112.

[14] Cope ED. On some extinct reptiles and Batrachia from the Judith River and Fox Hills beds of Montana. Proc Acad Nat Sci Phila. 1876;28: 340–359.

[15] Cope ED. The horned Dinosauria of the Laramie. Amer Nat. 1889; 23: 715–717.

[16] Penkalski P, Dodson P. The morphology and systematics of *Avaceratops*, a primitive horned dinosaur from the Judith River Formation (Late Campanian) of Montana, with the description of a second skull. J Vert Paleontol. 1999;19: 692–711.

[17] Leidy J. Notice of remains of extinct reptiles and fishes, discovered by Dr. FV Hayden in the badlands of the Judith River, Nebraska Territory. Proc Acad Nat Sci Philadelphia. 1856;8: 72–73.

[18] Cope ED. Descriptions of some vertebrate remains from the Fort Union beds of Montana. Proc Acad Nat Sci Philadelphia. 1876; 28: 248–261.

[19] Fiorillo AR, Currie PJ. Theropod teeth from the Judith River Formation (Upper Cretaceous) of south-central Montana. J Vert Paleontol. 1994;14: 74–80.

[20] Fiorillo AR. Taphonomy and depositional setting of Careless Creek Quarry (Judith River Formation), Wheatland County, Montana, USA. Palaeogeogr, Palaeoclimatol, Palaeoecol. 1991; 81: 281–311.

[21] Freedman Fowler EA, Horner JR. A new brachylophosaurin hadrosaur (Dinosauria: Ornithischia) with an intermediate nasal crest from the Campanian Judith River Formation of northcentral Montana. PLOS ONE. 2015;10(11): e0141304 10.1371/journal.pone.0141304 26560175 PMC4641681

[22] Schott RK, Evans DC, Williamson TE, Carr TD, Goodwin MB. The anatomy and systematics of *Colepiocephale lambei* (Dinosauria: Pachycephalosauridae). J Vert Paleontol. 2009;29: 771–786.

[23] Goodwin MB, Deino AL. The first radiometric ages from the Judith River Formation (Upper Cretaceous), Hill County, Montana. Can J Earth Sci. 1989;26: 1384–1391.

[24] Eberth DA, Hamblin AP. Tectonic, stratigraphic, and sedimentologic significance of a regional discontinuity in the upper Judith River Group (Belly River wedge) of southern Alberta, Saskatchewan, and northern Montana. Can J Earth Sci. 1993;30: 174–200.

[25] Mallon JC, Evans DC, Ryan MJ, Anderson JS. Megaherbivorous dinosaur turnover in the Dinosaur Park Formation (upper Campanian) of Alberta, Canada. Palaeogeogr, Palaeoclimatol, Palaeoecol. 2012;350: 124–138.

[26] Roberts EM, Deino AL, Chan MA. 40 Ar/39 Ar age of the Kaiparowits Formation, southern Utah, and correlation of contemporaneous Campanian strata and vertebrate faunas along the margin of the Western Interior Basin. Cretaceous Res. 2005; 26: 307–318.

[27] Roberts EM, Sampson SD, Deino AL, Bowring SA, Buchwaldt R. The Kaiparowits Formation: A remarkable record of Late Cretaceous terrestrial environments, ecosystems, and evolution in western North America In: Titus AL, Loewen MA, editors. At the Top of the Grand Staircase: the Late Cretaceous of Southern Utah. Bloomington, Indiana University Press; 2013, pp. 85–106.

[28] Sampson SD, Loewen MA. Unraveling a radiation: a review of the diversity, stratigraphic distribution, biogeography, and evolution of horned dinosaurs (Ornithischia: Ceratopsidae) In: Ryan MJ, Chinnery-Allgeier BJ, Eberth DA, editors. New perspectives on horned dinosaurs: the Royal Tyrrell Museum Ceratopsian Symposium; 2010 pp. 405–427.

[29] Shipman P. Life history of a fossil: an introduction to taphonomy and paleoecology. Cambridge: Harvard University Press; 1981.

[30] Lyman RL. Vertebrate taphonomy. Cambridge: Cambridge University Press; 1994.

[31] Britt BB, Eberth DA, Scheetz RD, Greenhalgh BW, Stadtman KL. Taphonomy of debris-flow hosted dinosaur bonebeds at Dalton Wells, Utah (Lower Cretaceous, Cedar Mountain Formation, USA). Palaeogeogr, Palaeoclimatol, Palaeoecol. 2009;280: 1–22.

[32] Ebeth DA, Getty MA. Ceratopsian bonebeds: occurrence, origins, and significance In: Currie PJ, Koppelhus EB, editors. Dinosaur Provincial Park: A Spectacular Ancient Ecosystem Revealed. Bloomington: Indiana University Press; 2005 pp. 501–536.

[33] Lamm E-T. Preparation and section of specimens In: Padian K, Lamm E-T, ediors. Bone histology of fossil tetrapods: advancing methods, analysis, and interpretation. Berkeley: University of California Press; 2013 pp. 55–160.

[34] Brown CM, Henderson DM. A new horned dinosaur reveals convergent evolution in cranial ornamentation in Ceratopsidae. Curr Biol. 2015;25:1641–1648. 10.1016/j.cub.2015.04.041 26051892

[35] Sampson SD, Loewen MA, Farke AA, Roberts EM, Forster CA, Smith JA, et al New horned dinosaurs from Utah provide evidence for intracontinental dinosaur endemism. PLOS One. 2010;5(9): e12292 10.1371/journal.pone.0012292 20877459 PMC2929175

[36] Mallon JC, Holmes R, Anderson JS, Farke AA, Evans DC. New information on the rare horned dinosaur *Arrhinoceratops brachyops* (Ornithischia: Ceratopsidae) from the Upper Cretaceous of Alberta, Canada. Can J Earth Sci. 2014;51: 618–634.

[37] Campbell JA, Ryan MJ, Holmes RB, Schröder-Adams CJ. A re-evaluation of the chasmosaurine ceratopsid genus *Chasmosaurus* (Dinosauria: Ornithischia) from the Upper Cretaceous (Campanian) Dinosaur Park Formation of western Canada. PLOS ONE. 2016;11(1): e0145805 10.1371/journal.pone.0145805 26726769 PMC4699738

[38] Hennig W. Phylogenetic systematics. Chicago: University of Chicago Press; 1999.

[39] Holmes RB. The postcranial skeleton of *Vagaceratops irvinensis* (Dinosauria, Ceratopsidae). Vert Anat Morphol Palaeontol. 2014;1: 1–21.

[40] Swofford DL. PAUP*. Phylogenetic analysis using parsimony (* and other methods), Version 4; Sunderland, Massachusetts: Sinauer Associates; 2003.

[41] Sorenson MD, Franzosa EA. TreeRot, Version 3 Boston: Boston University; 2007.

[42] Godfrey SJ, Holmes R. Cranial morphology and systematics of *Chasmosaurus* (Dinosauria: Ceratopsidae) from the Upper Cretaceous of western Canada. J Vert Paleontol. 1995; 15: 726–742.

[43] Lehman TM. The ceratopsian subfamily Chasmosaurinae: sexual dimorphism and systematics In: Currie PJ, Carpenter K, editors. Dinosaur systematics: approaches and perspectives; 1990 pp. 211–230.

[44] Maidment SCR, Barrett PM. A new specimen of Chasmosaurus belli (Ornithischia: Ceratopsidae), a revision of the genus, and the utility of postcrania in the taxonomy and systematics of ceratopsid dinosaurs. Zootaxa. 2011;2963(1): 1–47.

[45] Forster CA. New information on the skull of *Triceratops*. J Vert Paleontol. 1996;16: 246–58.

[46] Hatcher JB, Osborn HF, Marsh OC. The Ceratopsia. Monogr US Geol Surv. 1907;49: 1–300.

[47] Tanke DH, Farke AA. Bone resorption, bone lesions and extra cranial fenestrae in ceratopsid dinosaurs: a preliminary assessment In: Carpenter K, editor. Horns and beaks: ceratopsian and ornithopod dinosaurs. Bloomington: Indiana University Press; 2007 pp. 319–347.

[48] Edmund GA. On the special foramina in the jaws of many ornithischian dinosaurs. Life Sci Contrib, R Ont Mus. 1957;48: 1–14.

[49] Farke AA. Evolution, homology, and function of the supracranial sinuses in ceratopsian dinosaurs. J Vert Paleontol. 2010;30: 1486–1500.

[50] Farke AA. Morphology and ontogeny of the cornual sinuses in chasmosaurine dinosaurs (Ornithischia: Ceratopsidae). J Paleo. 2006;80: 780–785.

[51] Tanke DH, Rothschild BM. Paleopathologies in Albertan ceratopsids and their behavioral significance In: Ryan MJ, Chinnery-Allgeier BJ, Eberth DA, editors. New perspectives on horned dinosaurs: the Royal Tyrrell Museum Ceratopsian Symposium; 2010 pp. 355–84.

[52] Brown CM, Russell AP, Ryan MJ. Pattern and transition of surficial bone texture of the centrosaurine frill and their ontogenetic and taxonomic implications. J Vert Paleontol. 2009;29: 132–141.

[53] Ostrom JH. A functional analysis of jaw mechanics in the dinosaur *Triceratops*. Postilla. 1964;88: 1–35.

[54] Holliday CM. New insights into dinosaur jaw muscle anatomy. Anat Rec. 2009;292: 1246–1265. 10.1002/ar.20982 19711458

[55] Lehman TM. New data on the ceratopsian dinosaur *Pentaceratops sternbergii* Osborn from New Mexico. J Paleontol. 1993;2: 279–288.

[56] Longrich NR. *Mojoceratops perifania*, a new chasmosaurine ceratopsid from the late Campanian of western Canada. J Paleontol. 2010;84: 681–694.

[57] Mallon JC, Holmes R, Eberth DA, Ryan MJ, Anderson JS. Variation in the skull of *Anchiceratops* (Dinosauria, Ceratopsidae) from the Horseshoe Canyon Formation (Upper Cretaceous) of Alberta. J Vert Paleontol. 2011;31: 1047–1071.

[58] Rothschild BM, Martin LD. Skeletal impact of disease. New Mexico Mus Nat Hist Sci Bull. 2006;33: 1–226.

[59] Holmes RB, Forster C, Ryan M, Shepherd KM. A new species of *Chasmosaurus* (Dinosauria: Ceratopsia) from the Dinosaur Park Formation of southern Alberta. Can J Earth Sci. 2001;38: 1423–1438.

[60] Mallon JC, Anderson JS. Jaw mechanics and evolutionary paleoecology of the megaherbivorous dinosaurs from the Dinosaur Park Formation (upper Campanian) of Alberta, Canada. J Vert Paleontol. 2015;35(2): e904323.

[61] Mallon JC, Anderson JS. The functional and palaeoecological implications of tooth morphology and wear for the megaherbivorous dinosaurs from the Dinosaur Park Formation (upper Campanian) of Alberta, Canada. PLOS ONE. 2014;9(6): e98605. 24918431 10.1371/journal.pone.0098605 PMC4053334

[62] Holmes RB, Ryan MJ. The postcranial skeleton of *Styracosaurus albertensis*. Kirtlandia. 2013;58: 5–37.

[63] Ostrom JH, Wellnhofer P. The Munich specimen of *Triceratops* with a revision of the genus. Zitteliana. 1986;14: 111–158.

[64] Lull RS. A revision of the Ceratopsia or horned dinosaurs. Mem Peabody Mus Nat Hist. 1933;3(3): 1–175.

[65] Kozisek J, Derstler K. Scapular facets on the dorsal ribs of sauropod and neoceratopsian dinosaurs. J Vert Paleontol. 2004;24(3, Suppl): 80A.

[66] Larson P, Evans D, Ott C. Triceresies-a bold new look at *Triceratops*. J Vert Paleontol. 2004; 24(3, Suppl):82A.

[67] Mallon JC, Holmes R. Description of a complete and fully articulated chasmosaurine postcranium previously assigned to Anchiceratops (Dinosauria: Ceratopsia) In: Ryan MJ, Chinnery-Allgeier BJ, and Eberth DA, editors. New perspectives on horned dinosaurs: the Royal Tyrrell Museum Ceratopsian Symposium. Bloomington: Indiana University Press; 2010 pp. 189–202.

[68] Brown B. A complete skeleton of the horned dinosaur *Monoclonius*, and description of a second skeleton showing skin impressions. Bull Am Mus Nat Hist. 1917;37: 281–306.

[69] Wick SL, Lehman TM. A new ceratopsian dinosaur from the Javelina Formation (Maastrichtian) of West Texas and implications for chasmosaurine phylogeny. Naturwissenschaften. 2013;100: 667–682. 10.1007/s00114-013-1063-0 23728202

[70] Mallon JC, Ryan MJ, Campbell JA. Skull ontogeny in *Arrhinoceratops brachyops* (Ornithischia: Ceratopsidae) and other horned dinosaurs. Zool J Linn Soc. 2015;175: 910–929.

[71] Horner JR, Goodwin MB. Major cranial changes during *Triceratops* ontogeny. Proc R Soc B. 2006;273: 2757–2761. 17015322 10.1098/rspb.2006.3643 PMC1635501

[72] Longrich NR. The horned dinosaurs *Pentaceratops* and *Kosmoceratops* from the upper Campanian of Alberta and implications for dinosaur biogeography. Cretaceous Res. 2014;51: 292–308.

[73] Sternberg CM. The Edmonton fauna and description of a new Triceratops from the Upper Edmonton member; phylogeny of the Ceratopsidae. Natl Mus Can Bull. 1949; 113: 33–46.

[74] Langston W Jr. *Anchiceratops* from the Oldman Formation of Alberta. Natl Mus Can Nat Hist Pap. 1959; 3: 1–11.

[75] Sampson SD, Ryan MJ, Tanke DH. Craniofacial ontogeny in centrosaurine dinosaurs (Ornithischia: Ceratopsidae): taxonomic and behavioral implications. Zool J Linn Soc. 1997; 121: 293–337.

[76] Farke AA, Wolff ED, Tanke DH. Evidence of combat in *Triceratops*. PLOS ONE. 2009;4(1): e4252 10.1371/journal.pone.0004252 19172995 PMC2617760

[77] Krauss D, Pezon AN, Nguyen PE, Salame IS, Rywkin S. Evolutionary interactions between horn and frill morphology in chasmosaurine ceratopsians In: Ryan MJ, Chinnery-Allgeier BJ, and Eberth DA, editors. New perspectives on horned dinosaurs: the Royal Tyrrell Museum Ceratopsian Symposium. Bloomington: Indiana University Press; 2010 pp. 282–292.

[78] Leidy J. Extinct vertebrata from the Judith River and Great Lignite Formations of Nebraska. Trans Am Philos Soc. 1860;11: 139–154.

[79] Cope ED. Report on the geology of the region of the Judith River, Montana and on vertebrate fossils obtained on or near the Missouri River. Bull US Geol Geogr Surv Terr. 1877;19: 565–597.

[80] Sternberg CH. Notes on the fossil vertebrates collected on the Cope expedition to the Judith River and Cow Island beds, Montana, in 1876. Science. 1914;40: 134–135. 17776389 10.1126/science.40.1021.134

[81] Sampson SD, Loewen MA, Roberts EM, Getty MA. A new macrovertebrate assemblage from the Late Cretaceous (Campanian) of southern Utah In: Titus AL, Loewen MA, editors. At the top of the Grand Staircase: the Late Cretaceous of southern Utah. Bloomington: Indiana University Press; 2013 pp. 599–620.

[82] Horner JR. A new hadrosaur (Reptilia, Ornithischia) from the Upper Cretaceous Judith River Formation of Montana. J Vert Paleontol. 1988;8: 314–321.

[83] Evans DC, Ryan MJ. Cranial anatomy of *Wendiceratops pinhornensis* gen. et sp. nov., a centrosaurine ceratopsid (Dinosauria: Ornithischia) from the Oldman Formation (Campanian), Alberta, Canada, and the evolution of ceratopsid nasal ornamentation. PLoS ONE. 2015;10(7): e0130007 10.1371/journal.pone.0130007 26154293 PMC4496092

[84] Murphy NL, Trexler D, Thompson M. “Leonardo,” a mummified *Brachylophosaurus* from the Judith River Formation In: Carpenter K, editor. Horns and beaks: ceratopsian and ornithopod dinosaurs. Bloomington: Indiana University Press; 2006 pp. 117–133.

[85] Tweet JS, Chin K, Braman DR, Murphy NL. Probable gut contents within a specimen of *Brachylophosaurus canadensis* (Dinosauria: Hadrosauridae) from the Upper Cretaceous Judith River Formation of Montana. Palaios. 2008;23: 624–635.

[86] Holtz TR Jr. Tyrannosauroidea In: Weishampel DB, Dodson P, Osmólska H, editors. The Dinosauria. 2^nd^ ed Berkeley: University of California Press; 2004 pp. 111–136.

[87] Horner JR, Weishampel DB, Forster CA. Hadrosauridae In: Weishampel DB, Dodson P, Osmólska H, editors. The Dinosauria. 2^nd^ ed Berkeley: University of California Press; 2004 pp. 438–463.

[88] Makovicky PJ, Norell MA. Troodontidae In: Weishampel DB, Dodson P, Osmólska H, editors. The Dinosauria. 2^nd^ ed Berkeley: University of California Press; 2004 pp. 184–195.

[89] Horner JR. Egg clutches and embryos of two hadrosaurian dinosaurs. J Vert Paleontol. 1999;19: 607–611.

[90] Gates TA, Sampson SD, Zanno LE, Roberts EM, Eaton JG, Nydam RL, et al Biogeography of terrestrial and freshwater vertebrates from the Late Cretaceous (Campanian) Western Interior of North America. Palaeogeogr, Palaeoclimatol, Palaeoecol. 2010;291: 371–87.

[91] Gates TA, Prieto-Márquez A, Zanno LE. Mountain building triggered Late Cretaceous North American megaherbivore dinosaur radiation. PLOS ONE. 2012;7(8): e42135 10.1371/journal.pone.0042135 22876302 PMC3410882

[92] Longrich NR. *Titanoceratops ouranos*, a giant horned dinosaur from the late Campanian of New Mexico. Cretaceous Res. 2011;32: 264–276.

[93] Loewen MA, Farke AA, Sampson SD, Getty MA, Lund EK, O’Connor PM. Ceratopsid dinosaurs from the Grand Staircase of southern Utah In: Titus AL, Loewen MA, editors. At the top of the Grand Staircase: the Late Cretaceous of southern Utah. Bloomington: Indiana University Press; 2013 pp. 488–502.

