# Supplementary material for: Spiclypeus shipporum gen. et sp. nov., a Boldly Audacious New Chasmosaurine Ceratopsid (Dinosauria: Ornithischia) from the Judith River Formation (Upper Cretaceous: Campanian) of Montana, USA
Source: PLoS One. 2016 May 18;11(5):e0154218. doi: 10.1371/journal.pone.0154218 (PMC4871577; doi:10.1371/journal.pone.0154218)
Supplement: S1 Appendix — (DOCX) [file pone.0154218.s001.docx]

**S1 Appendix. Unambiguous synapomorphies uniting the ‘*Chasmosaurus*’ (blue), ‘*Triceratops*’ (red), and *Spiclypeus* (Vagaceratops + *Kosmoceratops*) clades shown in Fig 14 under different homology schemes.**

**Traditional homology scheme (Fig 14, top)**

‘*Chasmosaurus* clade’ (blue)

1. Nasals insert between premaxillae in dorsal view
2. Median parietal bar round to lenticular in cross-section
3. Dentary lateral ridge confluent with cutting surface of predentary absent

*Spiclypeus* + (*Vagaceratops* + *Kosmoceratops*) clade

1. External naris extends posteriorly over maxillary tooth row
2. Narial strut of premaxilla posteriorly inclined
3. Premaxillae insert between nasals in dorsal view
4. Postorbital horncore laterally curved in anterior view
5. Concave medial embayment of posterior parietal bar shallow, entire posterior bar is a V‐shaped embayment
6. Epiparietals fused to adjacent epiparietal at base
7. Epiparietal locus P1 strongly recurved triangular or recurved low gnarled triangular process
8. Epiparietal locus P2 strongly recurved triangular or recurved low gnarled triangular process
9. Epiparietal locus P2 recurved onto dorsal surface of frill

‘*Triceratops* clade’ (red)

1. External naris extends posteriorly over maxillary tooth row
2. Interpremaxillary fossa in premaxillary septum present
3. Epiparietal P1 oriented in the plane of the frill

**New homology scheme (Fig 14, bottom)**

‘*Triceratops* clade’ (red)

1. External naris extends posteriorly over maxillary tooth row
2. Interpremaxillary fenestra in premaxillary septum present
3. Posterior tip of posteroventral process of premaxilla intervenes between nasal and maxilla
4. Distal end of posteroventral process of premaxilla not forked
5. Accessory antorbital fenestra absent
6. Postorbital horncore centered posterodorsal to orbit, broad base with posterior margin of postorbital horncore extending well behind posterior orbit
7. Concave median embayment on posterior margin of parietal absent
8. Parietal fenestrae restricted to parietal
9. Anteroposterior thickness of parietal transverse bar at narrowest point broad, 20% or more of total parietal length
10. Median parietal bar relatively wide, transverse width 15% or more of total parietal length
11. Maximum proximodistal diameter of parietal fenestra 35% or less total parietal length
12. Episquamosals on midlateral squamosal margin large and elongate, greater than 90 mm long in adults
13. Epiparietal P0 present
14. Epiparietal P2 straight
