# Supplementary figures and images for: Spiclypeus shipporum gen. et sp. nov., a Boldly Audacious New Chasmosaurine Ceratopsid (Dinosauria: Ornithischia) from the Judith River Formation (Upper Cretaceous: Campanian) of Montana, USA
Source: PLoS One. 2016 May 18;11(5):e0154218. doi: 10.1371/journal.pone.0154218 (PMC4871577; doi:10.1371/journal.pone.0154218)

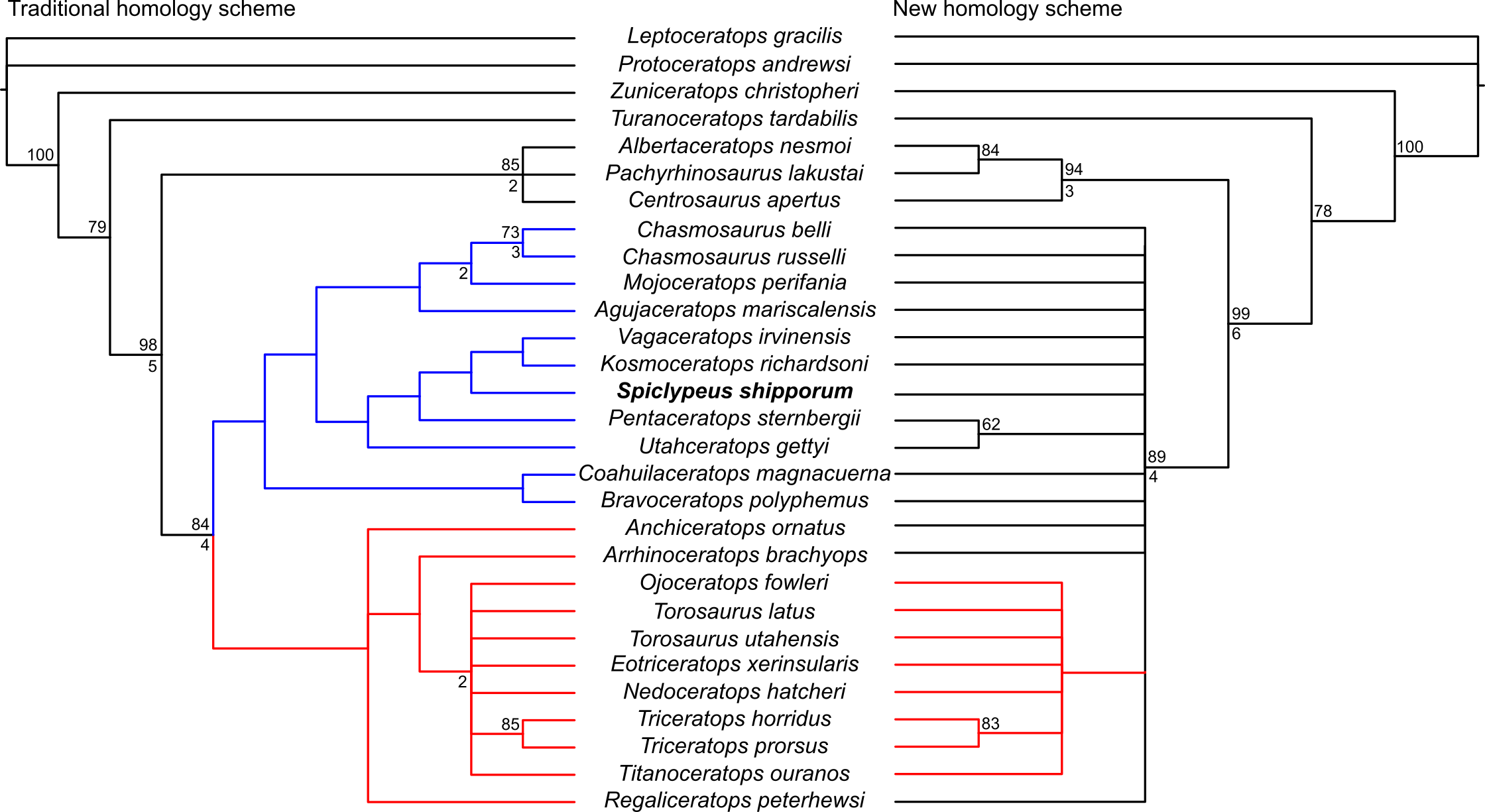

Supplement: S1 Fig — Left: strict consensus tree assuming traditional homology scheme for epiparietals (tree length = 332 steps, consistency index = 0.5873, retention index = 0.7345); right: strict consensus tree assuming new homology scheme for epiparietals (tree length = 439 steps, consistency index = 0.4487, retention index = 0.5346). Blue branches indicate ‘Chasmosaurus clade’ described in text; red branches indicate ‘Triceratops clade’. Numbers above nodes indicate bootstrap values >50%; numbers beneath nodes indicate Bremer support values >1. (TIF) [file pone.0154218.s002.tif]
