# Supplementary material for: Spiclypeus shipporum gen. et sp. nov., a Boldly Audacious New Chasmosaurine Ceratopsid (Dinosauria: Ornithischia) from the Judith River Formation (Upper Cretaceous: Campanian) of Montana, USA
Source: PLoS One. 2016 May 18;11(5):e0154218. doi: 10.1371/journal.pone.0154218 (PMC4871577; doi:10.1371/journal.pone.0154218)
Supplement: S1 File — (PDF) [file pone.0154218.s003.pdf]

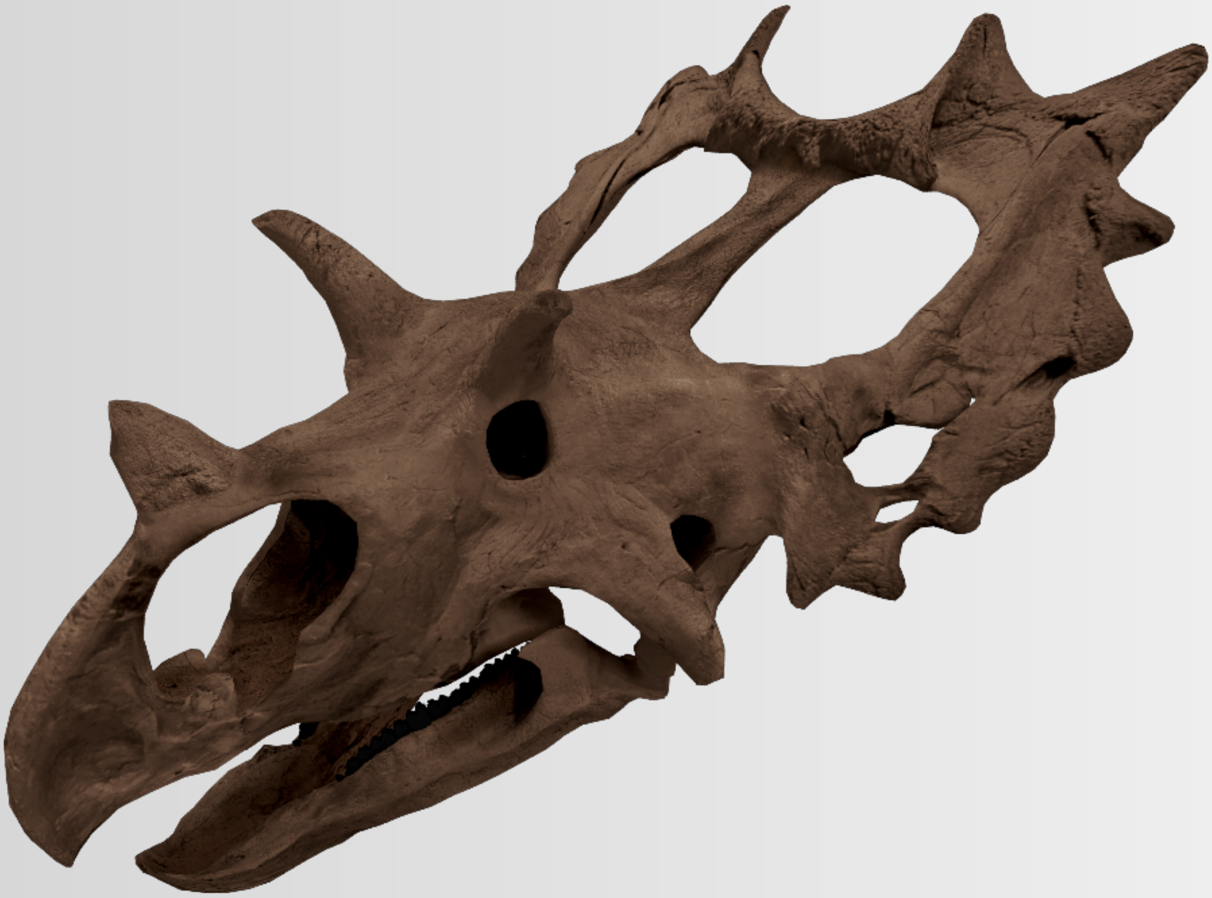

Skull reconstruction of *Spiclypeus shipporum* gen. et sp. nov. (CMN 57081).
Original reconstruction courtesy of Black Hills Institute of Geological Research, Inc.
